# The Dark and Gloomy Brain: Grey Matter Volume Alterations in Major Depressive Disorder–Fine-Grained Meta-Analyses

**DOI:** 10.1155/2024/6673522

**Published:** 2024-03-02

**Authors:** Zaira Romeo, Margherita Biondi, Leif Oltedal, Chiara Spironelli

**Affiliations:** ^1^Department of General Psychology, University of Padova, 35131 Padova, Italy; ^2^Padova Neuroscience Center, University of Padova, 35131 Padova, Italy; ^3^Department of Clinical Medicine, University of Bergen, 5020 Bergen, Norway; ^4^Mohn Medical Imaging and Visualization Centre, Department of Radiology, Haukeland University Hospital, 5021 Bergen, Norway

## Abstract

**Background:**

While the brain correlates of major depressive disorder (MDD) have been extensively studied, there is no consensus conclusion so far. Various meta-analyses tried to determine the most consistent findings, but the results are often discordant for grey matter volume (GMV) atrophy and hypertrophy. Applying rigorous and stringent inclusion criteria and controlling for confounding factors, such as the presence of anxiety comorbidity, we carried out two novel meta-analyses on the existing literature to unveil MDD signatures.

**Methods:**

A systematic literature search was performed up to January 2023. Seventy-three studies on MDD patients reporting GMV abnormalities were included in the first meta-analysis, for a total of 6167 patients and 6237 healthy controls (HC). To test the effects of anxiety comorbidity, we conducted a second meta-analysis, by adding to the original pure MDD sample a new cohort of MDD patients with comorbid anxiety disorders (308 patients and 342 HC). An activation likelihood estimation (ALE) analysis and a coordinate-based mapping approach separate for atrophy and hypertrophy were used to identify common brain structural alterations among patients.

**Results:**

The pure MDD sample exhibited atrophy in the left insula, as well as hypertrophy in the bilateral amygdala and parahippocampal gyri. When we added patients with comorbid anxiety to the original sample, bilateral insula atrophy emerged, whereas the hypertrophy results were not replicated.

**Conclusions:**

Our findings revealed important structural alterations in pure MDD patients, particularly in the insula and amygdala, which play key roles in sensory input integration and in emotional processing, respectively. Additionally, the amygdala and parahippocampal gyrus hypertrophy may be related to MDD functional overactivation to emotional stimuli, rumination, and overactive self-referential thinking. Conversely, the presence of anxiety comorbidity revealed separate effects which were not seen in the pure MDD sample, underscoring the importance of strict inclusion criteria for investigations of disorder-specific effects.

## 1. Introduction

Major depressive disorder (MDD) is the most typical condition among depressive disorders [1], where approximately 280 million individuals are affected worldwide [2]. Due to its pervasiveness and symptom severity, depression is nowadays a leading cause of disability and significantly contributes to the overall global burden of disease [3].

Decades of research have enabled the identification of numerous psychosocial, biological, and genetic correlates of MDD, but its pathophysiology remains unclear [4]. Nevertheless, the application of neuroimaging techniques such as structural magnetic resonance imaging (sMRI) to investigate the neural mechanisms underlying psychiatric diseases [5], together with the advances in automated procedures like voxel-based morphometry (VBM) which allows the objective evaluation of anatomical differences in a whole-brain manner [6], has been providing tangible evidence for the neurobiological correlates of MDD. In particular, grey matter volume (GMV) represents a straightforward and reliable measure for the investigation of brain morphometric differences. Using VBM, even focal and subtle differences can be identified between different populations (mainly patients vs. healthy individuals) [7]. Indeed, notwithstanding the emergence of more complex techniques, both the structural ones focusing on the connectome properties (e.g., [8, 9]) or the white matter fiber integrity (e.g., [10–12]) and those performing multimodal connectivity analyses (e.g., [13, 14]), studying GMV keeps providing foundational knowledge about brain organization and architecture, whereby its comprehension is essential for interpreting more complex structural and functional evidence that may involve multiple components. Moreover, structural aberrations in grey matter are likely to be associated with functional alterations, but the reverse process is more ambiguous: for example, impaired connectivity may occur in brains with normal-appearing GMV, as it is the case of disconnection syndromes [15]. For these reasons, the volumetric study of grey matter is still an informative method to assess brain changes in psychiatric illnesses, and MDD is one of those disorders where GMV investigation, and VBM, in particular, has contributed the most.

Several meta-analyses of whole-brain VBM studies have been published in the last decade, with the aim to summarize the brain structures associated with MDD [16–33]. Overall, these studies show a distribution of GMV atrophy across both cortical and subcortical regions, such as the cingulate [16, 18–21, 23–25, 27] and prefrontal cortices [16, 21, 23, 25, 32], frontal gyrus [18, 19, 21, 23, 24, 27, 29–31, 33], insula [21, 23, 24, 27, 29, 31], hippocampus [18, 21–24, 29, 30, 33], and parahippocampus [16, 19, 21, 29, 30, 33]. Interestingly, alterations in some of these areas have been associated to specific MDD symptoms, highlighting the role of MRI to study the relationship between brain changes and clinical characteristics of this disorder. For example, reduced hippocampal volume has been associated with deficits in memory performance [34] and with increased frequency of episodes or longer illness duration [35], while the cingulate cortex has been shown to be crucial in the regulation of affective states [36]. Moreover, dysfunctions in the insula—a region known for integrating information from limbic and frontal areas, with its anterior division particularly involved in cognitive and affective processes—have been suggested as a correlate for the lack of cognitive inhibition to negative emotions, rumination, and neuropsychological impairments commonly observed in MDD patients [37]. Findings are less consistent in the case of hypertrophy, where only a few whole-brain VBM meta-analyses revealed increased GMV in patients with respect to healthy controls and results were inconsistent across studies. For example, Wise et al. identified greater GMV within the bilateral superior occipital gyrus extending into the cuneus [21], while regions of increased volume were observed in the left visual cortex and the right temporoparietal junction by Sha and Banihashemi [25], or else in the right lingual gyrus of elderly patients as reported by Du et al. [33]. Among studies focusing on first-episode drug-naïve patients, the only convergent result is the GMV increase in the bilateral thalamus, whereas the other findings are still inconsistent [31, 32].

However, it should be pointed out that the above-mentioned results come from heterogeneous studies. Notably, most meta-analyses focused on samples including pharmacologically treated patients [16–26], further subdivided into responders and nonresponders by Liu et al. [27]. Conversely, some investigations considered first-episode cases [28, 29] or medication-free patients [30], whereas others considered only first-episode drug-naïve patients [31, 32]. A meta-analysis focusing only on late-life depression was also published [33]. Moreover, in some instances, broader selection criteria have been used: there are cases in which papers performing small volume correction—a violation of whole-brain analysis because it restricts the search area to a given region of interest—have been included (e.g., [22, 23]), or cases where other factors remained ambiguous, such as the lack of distinction between grey matter density or concentration and GMV (e.g., [18, 26]). Another issue worthy of attention is the presence of clinical samples with a spurious diagnostic profile, for example, by inclusion of remitted patients (e.g., [25]), subjects with a treatment-refractory condition (e.g., [24]), individuals suffering from secondary depression (e.g., [21]), or individuals who manifest subthreshold depressive symptoms (e.g., [19]).

When assessing the comparability of samples from different investigations, we found that patients with overt anxiety comorbidity were often not excluded. Indeed, despite numerous previous meta-analyses declaring a dual psychiatric diagnosis as an exclusion criterion, we still found traces of samples of MDD patients showing comorbid anxiety [17–21, 24, 25, 28, 30–32]. Although the link between anxiety and depression is undoubted [38] with estimated concurrency in about 46% of depressed individuals [39], anxiety and depression are distinct mental disorders, characterized by specific diagnostic criteria [1], and with different clinical and pharmacological treatments. As a consequence, anxiety disorders might have a specific role in affecting brain structures when co-occurring with MDD, and they should be treated like any other comorbid pathologies.

The present study is aimed at studying the brain correlates of MDD in its most characterizing symptomatology. Therefore, we conducted a novel meta-analysis of whole-brain VBM studies on a “pure” MDD sample (i.e., depressed patients without any comorbidities), carefully applying strict inclusion criteria in order to eliminate confounding factors as much as possible. However, anxious symptoms during MDD have been found in 50-60% of cases [40, 41]; hence, our pure MDD subjects were allowed to exhibit anxiety symptoms as long as the criteria for a secondary diagnosis were not fulfilled. Starting from this, we hypothesized that an overt comorbidity between MDD and an anxiety disorder might have a different impact than the simple co-occurrence of anxious symptoms during depressive episodes. This is particularly important considering that anxiety disorders have been associated with both shared and distinct structural alterations when compared with MDD. A comprehensive review of MRI studies revealed, among other brain structures, decreased volumes of the hippocampus, anterior cingulate cortex, insula, and amygdala in patients suffering from anxiety disorders [5]. The authors also highlighted how the variety of the etiopathogenesis among pathological subtypes and the presence of comorbidities can bias the results, preventing generalization [5]. Indeed, when focusing on subtypes of anxiety disorders—separate from MDD—specific alterations have appeared. For example, a recent whole-brain VBM meta-analysis found lower GMV in the thalamus and striatum of patients with social anxiety disorder, but reduced GMV in prefrontal and temporoparietal cortices, thalamus, striatum, and brainstem, together with increased GMV in temporo-parieto-occipital and prefrontal cortices of patients with panic disorder [42]. Therefore, to disclose any potential difference, we conducted a second meta-analysis by adding to the original pure MDD sample a group of MDD patients who also revealed a comorbidity to any of the DSM axis-I anxiety disorders.

The present work represents, to the best of our knowledge, the most complete and updated coordinate-based meta-analysis (CBMA) on MDD. All the studies on the matter published up to 31 January 2023, documenting alterations (both reduction and increase) of GMV at the whole-brain level, have been systematically searched, rigorously screened, and finally analyzed using an activation likelihood estimation (ALE) approach. The final aim is to improve our understanding of the pathophysiology of MDD, with an additional focus on the effects of co-occurrence of anxiety on brain grey matter.

## 2. Methods

### 2.1. Inclusion Criteria and Study Selection

A systematic and extensive literature search was carried out in PubMed before the end of January 2023 to identify potential studies according to the Preferred Reporting Items for Systematic Reviews and Meta-Analyses (PRISMA) guidelines [43].

The keywords used for the literature search were as follows: (1) (major depression OR major depressive disorder OR MDD) AND (voxel) AND (morphometry), (2) (major depression OR major depressive disorder OR MDD) AND (structural MRI OR sMRI), and (3) (major depression OR major depressive disorder OR MDD) AND (gray matter volume OR grey matter volume). The procedure returned 2111 records. Additionally, we inspected the complete reference lists of the previous meta-analyses on the matter [16–33]. This final check did not reveal other studies than those obtained with the PubMed search.

Studies were included if they met the following inclusion criteria: (1) they were published in peer-reviewed journals in English, (2) they compared MDD patients with healthy controls (HC), (3) they reported GMV abnormalities using sMRI (studies with no significant results were excluded, as well as studies investigating GM concentration or density), (4) they performed whole-brain VBM analysis (i.e., ROI or small volume correction analyses were not included), (5) they reported stereotactic coordinates in MNI or Talairach space (when the coordinates or the direction of the contrast between patients and HC was not clearly reported, we contacted the corresponding authors; in case of no response, these papers were excluded), (6) they did not investigate any kind of depression different from MDD (e.g., subthreshold depression, secondary depression, peripartum/postpartum depression, psychotic or bipolar depression, dysthymic disorder, or premenstrual dysphoric disorder; we also excluded patients in remission or in euthymic state and those who were treatment-resistant), and (7) they included patients with no overt physical, neurological, or psychic comorbidities. To ensure results belonging to a “pure” MDD sample, we first excluded patients with any comorbid anxiety disorders—but not those showing anxious symptoms. After that, we retrieved the discarded publications to examine the effect produced by adding patients with both disorders to the original sample.

**Figure 1:**
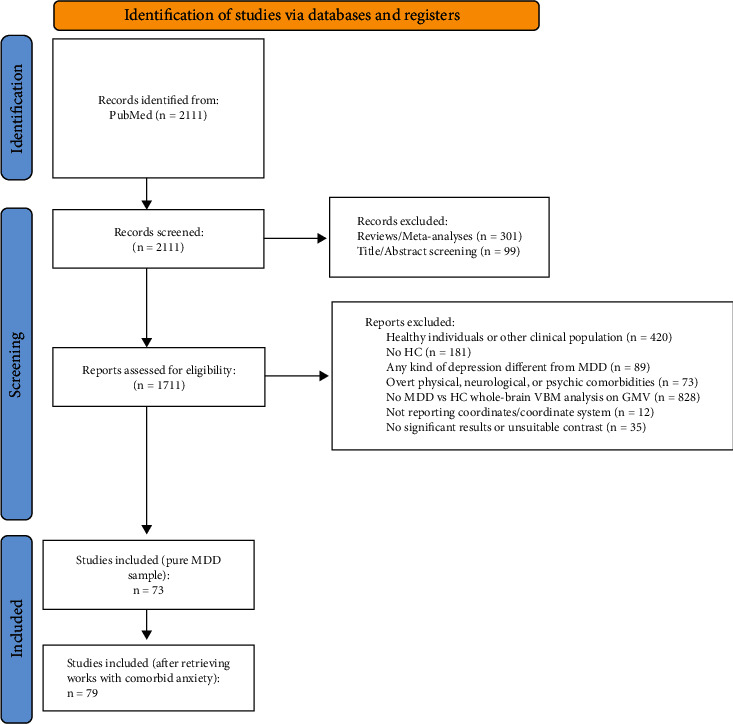
PRISMA flow diagram of study inclusion [43]. Abbreviations: GMV = grey matter volume; HC = healthy controls; MDD = major depressive disorder; VBM = voxel-based morphometry.

To achieve maximum research coverage and a sample as representative as possible of the clinical population, we did not impose limitations on age (although 95% of the reports included in the final pool analyzed participants whose age was above 18 and below 65) or medication status. We also included studies performing different subgroup comparisons, as long as patient samples were not overlapping. Finally, in the case of longitudinal clinical trials, we considered only the pretreatment baseline (e.g., before patients underwent electroconvulsive therapy) results. The inclusion process is summarized in the flowchart (Figure 1).

### 2.2. Data Extraction

Three authors visually inspected the “methods” and “results” paragraphs of each article, working independently. At the end of the procedure, the authors compared the results and any discrepancy was resolved by consensus. Next, data were manually extracted from articles that met the inclusion criteria. Data extraction was performed independently, and the resulting file was compared before performing statistical analyses. The results converged.

The final pool of published reports that was included in the meta-analysis on pure MDD consisted of 73 studies, listed in Table 1. From these studies, we extracted data representing a total of 6167 patients and 6237 HC. Notably, the number of healthy individuals here reported was obtained by counting them only once in case of studies performing different patients' subgroup comparisons with the same HC group (e.g., [44]).

Selected studies reported either decreases or increases in GMV, or both in some cases. In 67 papers, GMV atrophy was reported in patients with respect to HC, and six of them [44, 89, 92, 96, 99, 112] found this result in two separate comparisons between different subgroups of patients versus the same HC group; thus, the total amount of contrasts for GMV atrophy was 73. In the case of GMV hypertrophy in patients compared to HC, 29 papers (23 of which also found atrophy) returned this result; no different subgroup comparisons were performed; thus, the final number of included contrasts was 29.

To test the effects of anxiety comorbidity, we retrieved studies based on samples with dual diagnosis (*n* = 6) and an additional subsample affected by anxious comorbidity present in Qi et al. [64] previously discarded (Table 1). Therefore, we conducted a second meta-analysis adding to the original pure MDD sample a new cohort of 308 MDD patients with comorbid anxiety and 342 HC, therefore including six additional contrasts for GMV atrophy (*n* = 79) and two for GMV hypertrophy (*n* = 31). Therefore, this overall group, now labelled “MDD+A,” consisted of 6475 patients and 6579 healthy individuals.

### 2.3. Meta-Analyses

In compliance with the guidelines by Müller et al. [123], statistical analyses were run via GingerALE software version 3.0.2 [124, 125]. We carried out two different meta-analyses, the first one on the pure MDD sample and the second one on the MDD+A sample. Both of them were in turn subdivided into two meta-analyses performed separately, one for GMV atrophy and the other for GMV hypertrophy of patients compared to HC.

To weight study contributions, GingerALE uses sample sizes and coordinates, which must be expressed in the same stereotactic space; therefore, the first step consisted in converting Talairach coordinates into MNI space using the “convert foci” option provided in the GingerALE interface. Then, the activation likelihood estimation (ALE) method [126] was performed under the software to quantitatively assess the interstudy concordance. The ALE approach assesses the spatial convergence of reported coordinates across the experiments against the null hypothesis that the findings follow a random spatial distribution. The coordinates, or foci, are treated as three-dimensional Gaussian probability distributions centered at the given coordinates to generate per-experiment modeled atrophy/hypertrophy maps, which are subsequently joined in a union map [124, 127]. For each Gaussian distribution, the algorithm derives full-width half-maximum by considering the sample size of every single study. Finally, ALE tests for above-chance spatial convergence through a range of available thresholding options. In our meta-analyses, we set a statistical ALE map threshold for significance using cluster-level family-wise error (FWE) correction at *p* < 0.05 (5000 permutations), with cluster-forming threshold of *p* < 0.01. Forasmuch as each coordinate referred to the contrast between two groups (patients vs. healthy controls), the analysis relied on the *n* of the smaller of the two samples to yield a more conservative activation likelihood estimation [128].

## 3. Results

### 3.1. Patients' Characteristics

Patients included in the pure MDD sample (*n* = 6167, 60.2% females) were 34.9 years old on average, the diagnosis of major depression was mostly based on the DSM criteria [1] (the remaining used the ICD [129]), and they had no overt physical, neurological, or psychiatric comorbidities. The severity of depression was assessed with Hamilton Rating Scale for Depression (HAMD) [130] in most cases, but also Beck Depression Inventory (BDI) [131], Montgomery-Asberg Depression Rating Scale (MADRS) [132], and Zung's Self-Rating Depression Scale (SDS) [133] were sometimes administered. In the 18 included studies, anxious symptomatology—which did not reach the diagnostic threshold for an anxiety disorder diagnosis—was present too and typically measured with Hamilton Anxiety Rating Scale (HAMA) [134]; other scales used were Beck Anxiety Inventory (BAI) [135], Zung's Self-Rating Anxiety Scale (SAS) [136], and State-Trait Anxiety Inventory (STAI) [137]. During the course of illness, 1222 patients were drug-naïve, 1343 were drug-free (The *drug-naïve* patients have no prior history of drug treatment -most are first-episode patients- whereas the *drug-free* patients also include those who received pharmacological treatment in the past but were untreated at the time of the structural MRI acquisition -including, for example, patients who had suspended therapy or were in a “washout” condition), and 2284 were medicated; for the remaining patients, no information about drug status was available. In general, healthy controls were matched to the depressed patients demographically. Indeed, the HC recruited in the comparison studies (*n* = 6237) were similar to MDD patients regarding age (mean HC age = 34.11) and sex distribution (57% females).

Patients with comorbid anxiety added in the second meta-analysis (*n* = 308, 62% females) were 36.9 years old on average, and they all met the DSM criteria. Any other comorbidity was excluded. Their depressive symptomatology was mainly assessed with HAMD, while the scales used to evaluate the anxiety symptom severity were HAMA, SAS, and Panic Disorder Severity Scale (PDSS) [138]. The medication status was heterogeneous: in two studies, patients were drug-naïve, in three others drug-free, and in the last two under current treatment. The demographics of healthy subjects (*n* = 342) and MDD+A individuals were similar (mean HC age = 36.43, sex distribution = 64.6% females). See Table 1 for more detailed info.

### 3.2. Grey Matter Volume Changes in Pure MDD Patients versus Healthy Controls

**Figure 2:**
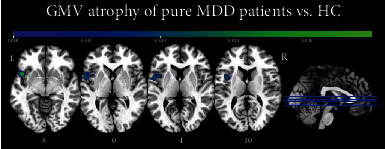
Results obtained from the meta-analysis focused on grey matter volume loss in pure MDD patients compared to HC.

The first meta-analysis was carried out on 73 contrasts (derived from 67 papers) that included 407 foci and compared 5509 pure MDD patients showing GMV atrophy versus 6618 HC (here, healthy individuals were counted twice in case of contrasts between two different subgroups of patients and the same HC group, as each contrast is taken into account separately). The minimum size for a cluster to be considered statistically significant was 2000 mm^3^. The minimum cluster size considered significant in the analyses is automatically calculated by the software GingerALE using the thresholding algorithm of cluster-level inference, which simulates random datasets using the characteristics of the original dataset: number of foci, number of foci groups, and subject sizes. By setting a threshold of *p* < 0.01, GingerALE finds above that threshold the contiguous volumes, “clusters,” and tracks the distribution of their volume. The cluster-level inference-corrected threshold sets the cluster minimum volume such that only, for example, 5% of the simulated data's clusters exceed this size. In other words, cluster-level inference uses a cluster forming threshold (*p* < 0.01) and the distribution of cluster sizes above the threshold to choose a minimum cluster size. Our results revealed a region of convergence of 3064 mm^3^ centered in the left insula (MNI coordinates: *X* = −47.3, *Y* = 9.1, *Z* = −1.7), with four peaks, three of which were located in the left insula (corresponding to Brodmann area (BA) 13) and one in the left superior temporal gyrus (BA 22). The maximum ALE value (0.0341, *p* < 0.001; *z* = 5.54) was found within the left insula (MNI coordinates: *X* = −46, *Y* = 12, *Z* = −8).  Table 2 provides a summary of all significant results.  Figure 2 shows that the significant cluster was lateralized in the left hemisphere and that it included the insula and the superior temporal gyrus.

**Figure 3:**
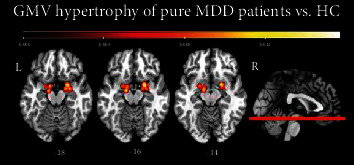
Results obtained from the meta-analysis focused on grey matter volume increase in pure MDD patients compared to HC.

Secondly, we meta-analyzed 29 contrasts (derived from 29 papers), for a total of 63 foci, in which 2188 pure MDD patients had higher GMV than 3072 HC. The minimum cluster size chosen to be statistically significant was 1680 mm^3^, and two regions converged above this threshold. The first cluster of 1696 mm^3^ was centered in the left parahippocampal gyrus (MNI coordinates: *X* = −14.7, *Y* = −4.2, *Z* = −15.5) with three peaks, all of them belonging to the left parahippocampal gyrus (BA 28/BA 34). The maximum ALE value (0.0121, *p* < 0.001; *z* = 3.84) was found within the left parahippocampal gyrus (MNI coordinates: *X* = −14, *Y* = −8, *Z* = −16). The second cluster of 1688 mm^3^ was centered in the right parahippocampal gyrus (MNI coordinates: *X* = 22.4, *Y* = −1.3, *Z* = −16.8) with three peaks (right BA 28/BA 34 and right amygdala), and the maximum ALE value (0.0156, *p* < 0.001; *z* = 4.38) was found within the right parahippocampal gyrus (MNI coordinates: *X* = 22, *Y* = 2, *Z* = −16). A summary of all significant results is provided in Table 3, whereas Figure 3 shows the significant clusters including the left and right parahippocampal gyri and amygdala.

### 3.3. Grey Matter Volume Changes in MDD+A Patients versus Healthy Controls

**Figure 4:**
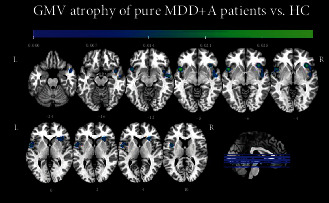
Results obtained from the meta-analysis focused on grey matter volume loss in MDD+A patients compared to HC.

This meta-analysis was conducted on 79 contrasts (derived from 73 papers) including 442 foci and comparing 5797 MDD+A patients showing reduced GMV versus 6960 HC (also here, healthy individuals were counted twice in case of contrasts between two different subgroups of patients and the same HC group, as each contrast is taken into account separately). The minimum size for a cluster to be considered statistically significant was 2304 mm^3^. The results showed two regions of convergence. The first cluster, 4216 mm^3^ in size, was centered in the right insula (MNI coordinates: *X* = 42.6, *Y* = 10.8, *Z* = −7.1) and had eight peaks distributed among the insula (BA 13), superior temporal gyrus (BA 21), temporal lobe (BA 38), and claustrum, all in the right hemisphere. The maximum ALE value (0.027, *p* < 0.001; *z* = 4.65) was located in the right insula (MNI coordinates: *X* = 38, *Y* = 22, *Z* = −4). The second cluster was 2952 mm^3^ and centered in the left insula (MNI coordinates: *X* = −47.9, *Y* = 8.8, *Z* = −1.8) with four peaks, three of which localized in the left insula (BA 13) and one in the left superior temporal gyrus (BA 22). The maximum ALE value (0.0341, *p* < 0.001; *z* = 5.47) was found in the left insula (MNI coordinates: *X* = −46, *Y* = 12, *Z* = −8).  Table 4 lists all significant peaks, and Figure 4 displays the significant clusters.

A second meta-analysis on 2238 MDD+A patients showing higher GMV compared to 3148 HC was conducted, comprising 31 contrasts (derived from 31 papers) for a total of 73 foci, but in this case, no statistically significant clusters were found.

Finally, an exploratory analysis on the six experiments in which patients with comorbid MDD and one overt anxiety disorder showed GMV atrophy when compared with HC has been carried out (see Supplementary materials). We did not consider the cases of hypertrophy due to the even more exiguous number.

## 4. Discussion

In the present CBMA study, four independent meta-analyses were performed, two of which focused on GMV atrophy and the other two on GMV hypertrophy, with the primary aim of clarifying the specific mechanisms underlying MDD in its purest symptomatologic manifestation and, as a complementary analysis, unveiling the critical role of comorbid anxiety. This last analysis is essential to disclose whether comorbid anxiety has a different impact on MDD brain structures than the simple co-occurrence of anxious symptoms during depressive episodes.

For our main analysis, the rigorous application of strict inclusion criteria permitted us to carry out a greater control of several confounding factors which have often been neglected in previous meta-analyses. Specifically, this includes the investigation of GMD or GMC rather than GMV (it is good practice to keep these values separate [139]) and the inclusion of patients suffering from a form of depression different from MDD, or affected by anxiety comorbidity.

The analysis of our pure MDD sample and similar healthy adults revealed two important findings: depressive patients exhibited both GMV atrophy and hypertrophy.

### 4.1. MDD Patients' Atrophy

Our meta-analysis of GMV loss included data from more than 5500 pure MDD patients and revealed an atrophic region centered in the left insula. Although numerous previous whole-brain meta-analyses [16–33] identified GMV atrophy in depressed patients, some of their results were discordant. These inconsistencies might be related to less stringent inclusion criteria, with the consequent presence of spurious samples with anxiety comorbidity. In fact, when we added patients with comorbid anxiety to our pure MDD sample, *bilateral* insula atrophy emerged. Interestingly, as GingerALE software reveals which studies contribute to the results, we noticed that one-third of the studies contributing to the right insula hub included patients with comorbidities. In other words, although the sample of patients with comorbidities was only 11% as large as the pure MDD sample (288/5509), their contribution was proportionally very large since the right insula hub did not emerge in the pure MDD sample. In contrast, only one of the studies contributing to the left hub belonged to the MDD+A sample. Considering these ratios and the fact that the left insula cluster was the main atrophic result of our pure MDD meta-analysis, we suggest that the left insula might predominately be affected in MDD patients, whereas the right insula might be less specific of MDD and rather associated with comorbid anxiety disorder. Furthermore, the exploratory analysis carried out on the small sample of MDD with anxiety disorder comorbidity revealed significant GMV atrophy in the right parahippocampal/subcallosal gyrus (see Table S1 and Figure S1 in the supplementary materials). This finding should be interpreted with caution, considering the very limited sample size of patient and control groups in the exploratory analysis. However, this analysis also reveals a reduced GMV in a right region, supporting a possible association between atrophy of the right hemisphere circuits and anxiety disorder comorbidity in MDD patients.

Regardless of its hemispheric location, it is important to note that our results converged in finding atrophy in the insula. The insula has wide connections with several brain areas involved in a variety of cognitive processing (e.g., [140, 141]) and has central position in emotional responses [142]. However, the insula also participates in language, sensory-motor, decision-making, salience, and attentional processing (e.g., [143–145]). Furthermore, the insula special function of (external) sensory input integration to the emotional processing in the limbic system supports its integral role for interoception—i.e., the awareness of the internal bodily state. This process represents the mechanism underlying humans' ability to perceive themselves as something different from the surrounding environment, allowing people to be aware of themselves, and finally to distinguish the image of “self” from “not oneself” [146–148]. In particular, the anterior portion of the insula is critical for processing the emotional component of interoception awareness, mainly depending on its connection with limbic regions [149, 150].

Notably, a recent meta-analysis revealed that important structural dysfunctions in left insula and temporofrontal regions contributed to the severity and persistence of schizophrenia auditory verbal hallucinations [151]. Furthermore, an atypical functional activation of the right insula was found in both hallucinating schizophrenia patients [152] and euthymic bipolar patients [153]. Considering that language lateralization is a crystallized characteristic of the human brain [154], a reduced GMV of left insula could alter the whole network and contribute to the confusion in processing internally and externally generated sensory/emotional experiences, thus possibly explaining several symptoms characterizing the most severe psychiatric disorders (e.g., semantic anomalies, thought disorders, ruminations, and auditory hallucinations).

### 4.2. MDD Patients' Hypertrophy

To the best of our knowledge, this is the most complete and updated meta-analysis that compared pure MDD patients showing hypertrophy with respect to healthy controls. We found hypertrophy in the bilateral amygdala and parahippocampal gyri in the pure MDD sample. This result, although still limited, is the newest and most promising one, given the recent and growing interest in structural hypertrophy. Past relevant studies from the ENIGMA MDD Consortium provided evidence of subcortical brain atrophy in a region close to this hub, i.e., the hippocampus [35, 155, 156]. Notably, these analyses were all carried out focusing on specific ROIs, including a small cluster of subcortical brain regions, not following a whole-brain approach. In their recent review, Schmaal et al. found reduced hippocampal volume in MDD patients compared with controls, with a modest effect size [35]. Interestingly, hippocampal atrophy appeared related to long-lasting, persistent, and recurring forms of MDD, characterized by progressive worsening of the disorder, rather than to a premorbid vulnerability factor [35].

The involvement of these areas in major depression has been widely investigated, especially in light of their functional role: the parahippocampal gyrus and the amygdala are part of a circuitry where emotion and memory functions have a mutual modulatory effect [157]. Consistent with this, cognitive theories suggest that memory biases of depressed individuals (e.g., greater access to negative autobiographic material) may interfere with emotion regulation strategies, and vice versa [158]. In particular, the parahippocampal gyrus is a region of the limbic system that has a main role in episodic memory and information processing, with a direct involvement in elaborating visuospatial data [159]. Furthermore, the parahippocampal region is characterized by numerous connections—with frontal, parietal, and temporal areas—which explain its key involvement in high-level cognitive functions, such as the processing of contextual associations [160]. Among its network of connections, the parahippocampus has been also linked to the default mode system [161], whose role in depressive symptoms has received wide consensus [162]. Furthermore, the default mode network with its self-referential nature (i.e., excessive self-inspection and internal and external monitoring) has been suggested as a neural basis of rumination in MDD [163], in the form of repetitive thinking over minor past failings, self-loathing, or guilty preoccupations [1]. Interestingly, Cooney et al. found that the parahippocampal activity was higher in depressed patients than healthy individuals during rumination [164], whereas Zamoscik et al. found that greater connectivity between the parahippocampus and default mode network in MDD patients was related to a sadder mood and more ruminative thoughts [165]. A comprehensive hypothesis suggests that the parahippocampal gyrus might be hyperactivated in depression because of its key role in rumination and autobiographical memory—the highly self-referential part of episodic memory—with a specific focus on negative episodes, thereby leading to the negative-valence emotions typical of MDD [165].

Notably, also the amygdala has been associated with rumination [166] and overactive self-referential thinking [167], functional activities that fit with its main role in affective control [168]. The amygdala has been indeed recognized as a crucial component of the brain circuitry underlying emotion [169], and its specific hyperactivity elicited by negative emotions has long contributed to the investigation of its involvement in depression [170]. For example, numerous functional imaging studies have shown that depressed individuals exhibit exaggerated amygdala reactivity to negative stimuli (e.g., [171–173]) and that, compared to healthy controls, these responses are maintained for a longer period of time [167]. Together with this, the amygdala is a key hub in memory and learning processes, reward mechanisms, and social behavior [174], which are all aspects known to be impaired in MDD [175]. Notably, some previous studies reporting enlarged amygdalar volumes in depressed patients (e.g., [176–178]), comprising also first-episode or recent-onset individuals, mutually agreed in speculating that the enlargement might result from elevated resting metabolism and overactivation in response to emotional stimuli.

Interestingly, it has been shown that treatment-resistant patients often show a functional restoration of both the parahippocampal gyrus and the amygdala when treated with more invasive approaches (e.g., ECT) [179, 180]. We therefore wondered whether our hypertrophy results depend on drug response, but what emerged from a “post hoc quality check” of the GingerALE output is that most of the contributing studies included only drug-free patients. Contrary to what we expected, our results revealed that hypertrophy was not a secondary phenomenon to pharmacological treatment. Then, a highly speculative interpretation of our results is that patients' hypertrophy represents a biomarker of pure MDD and not of their treatment response. In other words, some subcortical circuits, mainly involved in negative-valence emotional processing, are hyperstimulated by the disease symptoms, thus leading to an increased GMV. In the present study, it was not possible to carry out an in-depth analysis to further distinguish between drug-free patients (i.e., patients who had suspended therapy or were in a washout condition) from drug-naïve patients (i.e., patients with no prior history of drug treatment)—and it was not the main purpose of the present work—because many studies did not clarify this aspect, others defined drug-naïve patients as drug-free, and still others carried out multicenter researches on patients with various drug status and provided limited (when present) information on patients' pharmacological condition. The possible key role of drugs on hypertrophy results also appears considering the MDD+A meta-analysis, in which only 50 patients with anxiety comorbidity were added. Although the number of comorbidity patients was small, the result obtained for the pure MDD sample was not replicated. As comorbidity patients usually undergo multiple drug treatments, as they are characterized by more severe clinical pictures, a possible interpretation is that the addition of spurious participants may obscure the effects that appear spontaneously in a group of pure ones.

### 4.3. Final Remarks

In conclusion, increasing the sample size might not necessarily make the analysis statistically more powerful, as it might add a level of variance capable of modifying the results and their interpretation. In our specific case, we suggest that including data that make the pure sample spurious may produce less reliable results. In fact, the presence of an overt comorbidity between MDD and anxiety (unlike the occurrence of anxious symptoms only) in the MDD+A meta-analyses may have produced effects not specifically attributable to major depression.

Our work highlights the importance of using rigorous and stringent inclusion criteria for participant enrollment, in particular considering MDD patients with anxious comorbidity. From a more general perspective, the present study corroborates the importance of relying on neuroimaging methodologies for investigating the neurobiological mechanisms that underlie psychiatric disorders, but also for their clinical applications in diagnosis and treatment. In particular, neuroimaging findings can help to redefine diagnostic boundaries [181] and may provide markers of prognosis and monitor therapies, in an attempt to identify neural system abnormalities characterizing treatment-relevant endophenotypes [182]. Finally, they can provide the rationale for the development of specific neurostimulation approaches [183]. For example, considering our specific results, insula might be proposed—as has already been for other psychiatric conditions [184]—as a possible target for brain stimulation in MDD patients thanks to advancements in technology that allow for noninvasive stimulation of deeper brain areas (such as the insula). As neurostimulation techniques have widely demonstrated their effectiveness for MDD [185] and insular alterations have been consistently found across MDD studies, suggesting their putative key role in depression's pathophysiology, patients could concretely benefit from an intervention of deep stimulation targeted to this region, within which repeated sessions may induce long-lasting synaptic plasticity. Therefore, future studies might consider investigating insular stimulation as a treatment for MDD.

The present study has some limitations. First, only six studies met all our inclusion criteria for MDD patients with anxiety comorbidities. However, they led to important changes in our results. By adding future studies, it might be possible to disambiguate pure MDD brain correlates from those influenced also by anxiety comorbidity. Second, in our work, we explored the contribution of drug status only qualitatively, mainly because it was not possible to distinguish between drug-free patients and drug-naïve patients. Future studies may clarify this aspect, investigating results depending on pharmacological effects from those specific to MDD. Third, GingerALE software utilizes a coordinate-based approach, and the datasets for atrophy and hypertrophy are considered in separate analyses. The inclusion of studies with no significant results (*n* = 24 out of 35 no results/unsuitable contrasts in Figure 1) is not allowed, preventing us from estimating the risk of publication bias.

Notwithstanding these limiting factors, a clear pattern of left insula atrophy and bilateral amygdala and parahippocampal gyrus hypertrophy in pure depressive patients are noteworthy results deserving attention for future studies that aim at shedding light on anatomical correlates of mental disorders.

## Figures and Tables

**Table 1:** Studies included in the meta-analysis.

Study	Sample size (MDD vs. HC)	Age (MDD vs. HC)	Sex (MDD vs. HC)	Drug status	Diagnosis	Depression severity mean (scale type)	Anxiety symptom severity mean (scale type)	Anxiety comorbidity	Atrophy	Hypertrophy	Coordinate system
Wagner et al., [45]	15 vs. 16	41.4 vs. 38.8	15f vs. 16f	Drug-free	DSM-IV	23.5 (HAMD), 28.1 (BDI)	NA	No	Yes	No	Talairach
Leung et al., [46]	17 vs. 17	45.5 vs. 45.8	17f vs. 17f	Medicated	ICD-10	29.7 (BDI)	NA	No	Yes	Yes	Talairach
Mak et al., [47]	17 vs. 17	45.5 vs. 45.8	17f vs. 17f	Medicated	ICD-10	29.7 (BDI)	NA	No	Yes	No	MNI
Zou et al., [48]	23 vs. 23	31.1 vs. 36.6	13f/10m vs. 13f/10m	Drug-naïve	DSM-IV	24.4 (HAMD)	NA	No	Yes	No	Talairach
Cheng et al., [49]	68 vs. 68	29.91 vs. 29.91	47f/21m vs. 47f/21m	Drug-naïve	DSM-IV	22.32 (HAMD)	16.65 (HAMA)	No	Yes	No	MNI
Hwang et al., [50]	70 vs. 26	79.4 vs. 79.5	70m vs. 26m	NA	DSM-IV	29.1 (HAMD)	NA	No	Yes	Yes	Talairach
Scheuerecker et al., [51]	13 vs. 15	37.9 vs. 35.5	3f/10m vs. 5f/10m	Drug-free	DSM-IV	20.5 (HAMD)	NA	No	Yes	Yes	MNI
Amico et al., [52]	33 vs. 30	32 vs. 30.7	14f/19m vs. 13f/17m	Medicated	NA	23 (HAMD)	NA	No	No	Yes	MNI
Salvadore et al., [53]	58 vs. 107	38.8 vs. 36.2	37f/21m vs. 60f/47m	Drug-free	DSM-IV	26 (MADRS)	NA	No	Yes	No	Talairach
Ma et al., [54]	17 vs. 17	26.71 vs. 24.24	7f/10m vs. 7f/10m	Drug-naïve	DSM-IV	25.58 (HAMD)	NA	No	Yes	No	MNI
Wang et al., [55]	18 vs. 18	34 vs. 35	9f/9m vs. 9f/9m	Drug-naïve	DSM-IV	25 (HAMD)	17 (HAMA)	No	Yes	No	MNI
Grieve et al., [56]	102 vs. 34	31.5 vs. 33.8	54f/48m vs. 16f/18m	Drug-free	DSM-IV	21 (HAMD)	NA	No	Yes	No	MNI
Chaney et al., [57]	37 vs. 46	39.75 vs. 40.25	21f/16m vs. 28f/18m	Medicated	DSM-IV	28.6 (HAMD), 34.1 (BDI)	NA	No	Yes	Yes	MNI
Guo et al., [58]	44 vs. 44	27.52 vs. 29.39	22f/22m vs. 24f/20m	Drug-naïve	DSM-IV	25.18 (HAMD)	NA	No	Yes	No	MNI
Jung et al., [59]	24 vs. 29	43 vs. 43.6	17f/7m vs. 21f/8m	Medicated	DSM-IV	20.9 (HAMD)	NA	No	Yes	No	MNI
Kong et al., [60]	28 vs. 28	34.42 vs. 32.07	17f/11m vs. 14f/14m	Drug-naïve	DSM-IV	21.65 (HAMD)	NA	No	Yes	Yes	MNI
Lai et al., [61]	38 vs. 27	36.57 vs. 38.29	20f/18m vs. 15f/12m	Drug-naïve	DSM-IV	22.26 (HAMD)	2.36 (HAMA)	No	Yes	No	MNI
Modinos et al., [62]	23 vs. 46	44.6 vs. 25.3	20f/3m vs. 14f/32m	NA	DSM-IV	NA	NA	No	Yes	No	MNI
Nakano et al., [63]	36 vs. 54	49 vs. 45.4	22f/14m vs. 27f/27m	Medicated	DSM-IV-TR	15.4 (HAMD)	NA	No	Yes	No	MNI
Peng et al., [44]	20 vs. 28	27.75 vs. 28.61	13f/7m vs. 13f/15m	Medicated	DSM-IV	25.95 (HAMD), 55.05 (SDS)	NA	No	Yes	Yes	MNI
	18 vs. 28	31.06 vs. 28.61	12f/6m vs. 13f/15m	Medicated	DSM-IV	24.06 (HAMD), 56.94 (SDS)	NA	No	Yes	No	MNI
Qi et al., [64]	18 vs. 28	31.06 vs. 28.61	11f/7m vs. 13f/15m	Drug-free	DSM-IV	22.28 (HAMD), 51.67 (SDS)	8.11 (HAMA), 27.67 (SAS)	No	Yes	No	MNI
Qiu et al., [65]	46 vs. 46	34.9 vs. 35.4	33f/13m vs. 33f/13m	Drug-naïve	DSM-IV	23.3 (HAMD)	NA	No	No	Yes	MNI
Cai et al., [66]	23 vs. 23	30 vs. 28.2	10f/13m vs. 10f/13m	Medicated	DSM-IV	29.7 (HAMD)	9.22 (HAMA)	No	Yes	No	MNI
Dannlowski et al., [67]	171 vs. 512	38.65 vs. 33.5	105f/66m vs. 289f/223m	Medicated	DSM-IV	23.6 (BDI)	59.35 (STAI-trait)	No	Yes	No	MNI
Fang et al., [68]	20 vs. 18	59.2 vs. 59.1	8f/12m vs. 8f/10m	Medicated	DSM-IV	26.6 (HAMD)	NA	No	Yes	No	MNI
Lai et al., [69]	53 vs. 54	40.07 vs. 40.38	28f/25m vs. 29f/25m	Drug-naïve	DSM-IV	22.43 (HAMD)	2.20 (HAMA)	No	Yes	No	Talairach
Vasic et al., [70]	43 vs. 29	37.1 vs. 34.5	26f/17m vs. 18f/11m	Medicated	DSM-IV	20.09 (HAMD), 29 (BDI)	NA	No	Yes	No	MNI
Watanabe et al., [71]	29 vs. 45	41.05 vs. 41.4	13f/16m vs. 12f/33m	Drug-free	DSM-IV-TR	21.05 (HAMD)	NA	No	Yes	No	Talairach
Yang et al., (a) [72]	50 vs. 50	31.12 vs. 31.30	31f/19m vs. 31f/19m	Drug-free	DSM-IV	23.10 (HAMD)	16.12 (HAMA)	No	Yes	Yes	MNI
Yang et al., (b) [73]	51 vs. 51	30.98 vs. 31.14	31f/20m vs. 31f/20m	Drug-free	DSM-IV	22.90 (HAMD)	16.18 (HAMA)	No	Yes	Yes	MNI
Chen et al., [74]	27 vs. 28	33 vs. 33	14f/13m vs. 14f/14m	Drug-naïve	DSM-IV	22 (HAMD)	NA	No	No	Yes	MNI
Opel et al., [75]	20 vs. 20	37.9 vs. 36.3	10f/10m vs. 10f/10m	Medicated	DSM-IV	22.2 (HAMD), 45.3 (BDI)	NA	No	Yes	No	MNI
Qiu et al., [76]	12 vs. 15	34.4 vs. 33.7	8f/4m vs. 10f/5m	Drug-free	DSM-IV	35.9 (HAMD)	NA	No	Yes	No	MNI
Shen et al., [77]	147 vs. 130	30.58 vs. 30.09	97f/50m vs. 81f/49m	Drug-naïve	DSM-IV	23.83 (HAMD)	NA	No	Yes	Yes	MNI
Wang et al., [78]	25 vs. 35	32.11 vs. 33.28	11f/14m vs. 16f/19m	Medicated	DSM-IV	29.32 (HAMD)	5.32 (HAMA)	No	Yes	No	MNI
Igata et al., [79]	27 vs. 44	45.8 vs. 41.2	12f/15m vs. 12f/32m	Drug-naïve	DSM-IV-TR	21.8 (HAMD)	NA	No	Yes	No	MNI
Yang et al., [80]	82 vs. 82	28.85 vs. 27.72	53f/29m vs. 53f/29m	Drug-free	DSM-IV-TR	23.1 (HAMD)	NA	No	Yes	No	MNI
Zhao et al., [81]	37 vs. 41	26.7 vs. 27.1	12f/25m vs. 15f/26m	Drug-naïve	DSM-IV	25 (HAMD)	28.1 (HAMA)	No	Yes	Yes	MNI
Zhuo et al., [82]	45 vs. 48	38.8 vs. 38.6	26f/19m vs. 27f/21m	Medicated	DSM-IV	27.8 (HAMD)	NA	No	Yes	No	MNI
Chang et al., [83]	108 vs. 156	20.61 vs. 22.25	70f/38m vs. 93f/63m	Medicated	DSM-IV	20.15 (HAMD)	14.94 (HAMA)	No	Yes	No	MNI
Chen et al., [84]	36 vs. 47	30.7 vs. 29.7	16f/20m vs. 25f/22m	Drug-free	DSM-IV	28 (HAMD)	NA	No	Yes	Yes	MNI
Lu et al., [85]	76 vs. 86	33.4 vs. 34.65	44f/32m vs. 43f/43m	Drug-free	DSM-IV	33.3 (HAMD)	NA	No	Yes	Yes	MNI
Zaremba et al., [86]	37 vs. 54	37 vs. 37.5	19f/18m vs. 24f/30m	Medicated	DSM-IV	22.9 (HAMD)	NA	No	Yes	No	MNI
Zhou et al., [87]	114 vs. 111	28.17 vs. 27.63	54f/60m vs. 58f/53m	Drug-free	DSM-IV-TR	No less than 17 (HAMD)	NA	No	Yes	No	MNI
Gong et al., [88]	92 vs. 201	34.96 vs. 29.61	49f/43m vs. 123f/78m	Drug-naïve	DSM-IV	23.06 (HAMD)	NA	No	No	Yes	MNI
Hellewell et al., [89]	98 vs. 66	33.3 vs. 30.09	45f/53m vs. 33f/33m	Drug-free	DSM-IV	21.1 (HAMD)	NA	No	Yes	No	MNI
	131 vs. 66	33.2 vs. 30.09	77f/54m vs. 33f/33m	Drug-free	DSM-IV	21.9 (HAMD)	NA	No	Yes	No	MNI
Kandilarova et al., [90]	39 vs. 42	47.7 vs. 42.6	29f/10m vs. 29f/13m	Medicated	DSM-IV-TR	29.1 (MADRS)	NA	No	Yes	No	MNI
Li et al., [91]	56 vs. 56	35.1 vs. 30.7	36f/20m vs. 23f/33m	Drug-naïve	DSM-IV-TR	25.1 (HAMD)	NA	No	Yes	Yes	MNI
Liu et al., [92]	24 vs. 30	34.79 vs. 33.43	15f/9m vs. 14f/16m	Drug-naïve	DSM-5	25.5 (HAMD)	NA	No	Yes	No	MNI
	21 vs. 30	34.14 vs. 33.43	11f/10m vs. 14f/16m	Drug-naïve	DSM-5	24.48 (HAMD)	NA	No	Yes	Yes	MNI
Peng et al., [93]	104 vs. 160	33 vs. 32	62f/42m vs. 93f/67m	NA	DSM-IV	28 (HAMD)	28 (HAMA)	No	Yes	Yes	MNI
	57 vs. 160	31 vs. 32	36f/21m vs. 93f/67m	NA	DSM-IV	24 (HAMD)	17 (HAMA)	No	No	Yes	MNI
Straub et al., [94]	42 vs. 43	NA vs. 17.62	NA vs. 38f/5m	Medicated	DSM-IV	NA	NA	No	No	Yes	MNI
Chen et al., [95]	22 vs. 22	28.7 vs. 27.4	18f/4m vs. 18f/4m	Medicated	DSM-5	24.5 (MADRS)	NA	No	Yes	No	MNI
Liu et al., [96]	22 vs. 27	32.09 vs. 32.44	12f/10m vs. 14f/13m	Drug-naïve	DSM-5	27.03 (HAMD)	NA	No	Yes	Yes	MNI
	36 vs. 27	35.75 vs. 32.44	21f/15m vs. 14f/13m	Drug-naïve	DSM-5	24.45 (HAMD)	NA	No	Yes	No	MNI
Meng et al., [97]	159 vs. 53	33.71 vs. 35.64	83f/76m vs. 25f/28m	Drug-naïve	DSM-5	23.6 (HAMD)	NA	No	Yes	No	MNI
Nan et al., [98]	166 vs. 166	33.69 vs. 34.64	115f/51m vs. 115f/51m	Drug-naïve	DSM-IV	23.75 (HAMD)	NA	No	Yes	Yes	MNI
Yang et al., [99]	68 vs. 103	32.8 vs. 32.1	54f/14m vs. 67f/36m	Drug-free	DSM-IV	24 (HAMD)	NA	No	Yes	Yes	MNI
	119 vs. 103	34.6 vs. 32.1	65f/54m vs. 67f/36m	Drug-free	DSM-IV	21.9 (HAMD)	NA	No	Yes	No	MNI
Zhang et al., [100]	53 vs. 50	38.1 vs. 34.2	34f/19m vs. 34f/16m	Drug-naïve	ICD-10	19.83 (HAMD)	NA	No	Yes	No	MNI
Jiang et al., [101]	20 vs. 30	27.4 vs. 26.47	12f/8m vs. 15f/15m	Drug-naïve	DSM-IV	21.25 (HAMD)	18.81 (HAMA)	No	Yes	No	MNI
Liu et al., [102]	149 vs. 446	30.76 vs. 33.15	93f/56m vs. 252f/194m	NA	DSM-IV	19.28 (HAMD)	NA	No	Yes	Yes	MNI
	335 vs. 446	34.35 vs. 33.15	219f/116m vs. 252f/194m	NA	DSM-IV	23.68 (HAMD)	NA	No	No	Yes	MNI
Ma et al., [103]	52 vs. 65	24.98 vs. 25.25	34f/18m vs. 32f/33m	Medicated	DSM-IV-TR	NA	NA	No	Yes	No	MNI
Takamiya et al., [104]	48 vs. 52	74.1 vs. 72.4	33f/15m vs. 37f/15m	Medicated	DSM-IV-TR	23.1 (Geriatric Depression Scale)	NA	No	Yes	No	MNI
Zhang et al., [105]	20 vs. 20	28 vs. 31.7	13f/7m vs. 10f/10m	Drug-free	DSM-IV	26.5 (HAMD)	NA	No	Yes	Yes	MNI
Zhou et al., [106]	109 vs. 163	27.43 vs. 28.1	72f/37m vs. 96f/67m	Drug-free	DSM-IV-TR	20.4 (HAMD)	16.22 (HAMA)	No	Yes	No	MNI
Kang et al., [107]	77 vs. 111	22.09 vs. 26.01	62f/15m vs. 87f/24m	Medicated	DSM-5	NA	NA	No	Yes	No	MNI
Li et al., [108]	30 vs. 25	14.6 vs. 15.48	22f/8m vs. 19f/6m	Drug-free	DSM-IV	29.03 (HAMD)	NA	No	Yes	No	MNI
Liu et al., [109]	64 vs. 61	28.47 vs. 30.49	37f/27m vs. 25f/36m	Drug-free	DSM-IV	27.78 (HAMD)	NA	No	Yes	No	MNI
Lu et al.,[110]	108 vs. 99	33.56 vs. 35.93	63f/45m vs. 50f/49m	Medicated	DSM-IV-TR	27.07 (HAMD)	NA	No	Yes	No	MNI
Lu et al., [111]	22 vs. 20	28.6 vs. 27.7	17f/5m vs. 10f/10m	Drug-free	DSM-IV-TR	25.2 (HAMD)	NA	No	Yes	No	MNI
Sun et al., [112]	342 vs. 510	35.98 vs. 33.24	221f/121m vs. 291f/219m	NA	DSM-IV or ICD-10	24.36 (HAMD)	NA	No	Yes	Yes	MNI
	208 vs. 510	31.24 vs. 33.24	131f/77m vs. 291f/219m	NA	DSM-IV or ICD-10	16.83 (HAMD)	NA	No	Yes	No	MNI
Wang et al., [113]	1082 vs. 990	37.23 vs. 37.45	678f/404m vs. 580f/410m	Medicated	DSM-5 or ICD-10	20.75 (HAMD)	19.09 (HAMA)	No	Yes	No	MNI
Yang et al., [114]	85 vs. 95	32.44 vs. 30.21	51f/34m vs. 48f/47m	Medicated	DSM-IV	18.6 (BDI)	42.83 (BAI)	No	Yes	No	MNI
Yu et al., [115]	35 vs. 53	30.26 vs. 27.47	20f/15m vs. 31f/22m	Medicated	DSM-IV	21.83 (HAMD)	NA	No	Yes	No	MNI
Zhang et al., [116]	26 vs. 35	26.42 vs. 25.45	13f/13m vs. 18f/17m	Drug-free	DSM-IV	21.12 (HAMD)	NA	No	No	Yes	MNI
Lai et al., [117]	16 vs. 15	37.91 vs. 34.3	11f/5m vs. 11f/4m	Drug-naïve	DSM-IV	35.91 (HAMD), 26.09 (QIDS-SR16)	33 (HAMA), 18.55 (PDSS)	Yes (16 patients with comorbid panic disorder)	Yes	No	MNI
Qi et al., [64]	20 vs. 28	28.65 vs. 28.61	9f/11m vs. 13f/15m	Drug-free	DSM-IV	20.25 (HAMD), 51.67 (SDS)	16.80 (HAMA), 47.10 (SAS)	Yes (patients with comorbid anxiety disorders)	No	Yes	MNI
Stratmann et al., [118]	132 vs. 132	37.86 vs. 37.82	76f/56m vs. 74f/58m	Medicated	DSM-IV	20.15 (HAMD), 21.95 (BDI)	NA	Yes (41 patients with comorbid anxiety disorders)	Yes	No	MNI
Harada et al., [119]	45 vs. 61	60.2 vs. 62.9	26f/19m vs. 44f/17m	Medicated	DSM-IV-TR	24.8 (BDI), 17.3 (SIGH-D)	NA	Yes (1 patient with comorbid panic disorder; 1 patient with comorbid social anxiety disorder)	Yes	No	MNI
Yang et al., [120]	35 vs. 23	44.54 vs. 39.09	35f vs. 23f	Drug-free	DSM-IV	28.29 (HAMD)	20.17 (HAMA)	Yes (patients with high rates of comorbid anxiety disorders)	Yes	No	MNI
Lu et al., [121]	30 vs. 48	23.95 vs. 21.5	13f/17m vs. 30f/18m	Drug-free	DSM-IV	30.1 (HAMD), 71.5 (SDS)	NA	Yes (patients with comorbid anxiety disorders)	Yes	Yes	MNI
Zhang et al., [122]	30 vs. 63	25 vs. 23	21f/9m vs. 39f/24m	Drug-naïve	DSM-5	26.5 (HAMD)	28 (HAMA)	Yes (patients with comorbid anxiety disorders)	Yes	No	MNI

Abbreviations: BAI = Beck Anxiety Inventory; BDI = Beck Depression Inventory; DSM = Diagnostic and Statistical Manual of Mental Disorders; HAMA = Hamilton Anxiety Rating Scale; HAMD = Hamilton Rating Scale for Depression; ICD = International Classification of Diseases; MADRS = Montgomery-Asberg Depression Rating Scale; QIDS-SR16 = Quick Inventory of Depressive Symptomatology; PDSS = Panic Disorder Severity Scale; SAS = Zung's Self-Rating Anxiety Scale; SDS = Zung's Self-Rating Depression Scale; SIGH-D = Structured Interview Guide for the Hamilton Depression Rating Scale; STAI = State-Trait Anxiety Inventory.

**Table 2:** Significant results of the four peaks belonging to the cluster of GMV atrophy in pure MDD patients versus healthy controls.

Cluster	Anatomical label	BA	MNI coordinates			Size (mm^3^)	ALE value	*p* value	*z* score
			*X*	*Y*	*Z*				
1	Left insula	13	-46	12	-8	3064	0.0340712	<0.00000001	5.543614
	Left superior temporal gyrus	22	-54	6	0		0.0178932	<0.00020437	3.534379
	Left insula	13	-40	4	10		0.0176861	<0.00022831	3.504988
	Left insula	13	-48	10	4		0.0132937	<0.00223949	2.842296

**Table 3:** Significant peaks' results of the two clusters of GMV hypertrophy in pure MDD patients versus healthy controls.

Cluster	Anatomical label	BA	MNI coordinates			Size (mm^3^)	ALE value	*p* value	*z* score
			*X*	*Y*	*Z*				
1	Left parahippocampal gyrus	28	-14	-8	-16	1696	0.0120923	<0.00006059	3.843722
	Left parahippocampal gyrus	34	-12	0	-18		0.0107584	<0.00013409	3.644233
	Left parahippocampal gyrus	34	-20	0	-14		0.0095657	<0.00043853	3.327254

2	Right parahippocampal gyrus	34	22	2	-16	1688	0.0156489	<0.00000596	4.37878
	Right parahippocampal gyrus, amygdala		26	-4	-18		0.0109239	<0.00012228	3.66787
	Right parahippocampal gyrus	28	18	-6	-18		0.0107988	<0.00013105	3.65012

**Table 4:** Significant peaks' results of the two clusters of GMV atrophy in MDD+A patients versus healthy controls.

Cluster	Anatomical label	BA	MNI coordinates			Size (mm^3^)	ALE value	*p* value	*z* score
			*X*	*Y*	*Z*				
1	Right insula	13	38	22	-4	4216	0.0269826	<0.00000166	4.649883
	Right superior temporal gyrus	21	56	-6	-12		0.0200830	<0.00008093	3.772142
	Right claustrum		26	24	2		0.0178103	<0.00027382	3.456300
	Right insula	13	48	-4	-6		0.0170445	<0.00041425	3.343091
	Right sublobar extranuclear	13	42	6	-16		0.0165700	<0.00053162	3.273233
	Right insula	13	48	16	-4		0.0157237	<0.00082873	3.145600
	Right insula	13	48	4	-8		0.0141346	<0.00182844	2.906337
	Right temporal lobe	38	50	6	-24		0.0130762	<0.00300305	2.747448

2	Left insula	13	-46	12	-8	2952	0.0340717	<0.00000002	5.466796
	Left superior temporal gyrus	22	-54	4	0		0.0182960	<0.00021031	3.526797
	Left insula	13	-40	4	10		0.0176863	<0.00029208	3.438860
	Left insula	13	-48	10	4		0.0132938	<0.00272476	2.779186

## Data Availability

The template data collection forms, data extracted from included studies, data used for all analyses, analytic code, and any other materials used in the meta-analyses can be requested to the corresponding author upon justified request for academic purposes only.

## Supplementary Materials

Supplementary Materials Grey matter volume atrophy in the subgroup of patients with comorbid MDD and anxiety: an exploratory analysis. We conducted an exploratory analysis on the six experiments in which patients with comorbid MDD and one overt anxiety disorder showed GMV atrophy when compared with HC; we did not consider the cases of hypertrophy due to the even more exiguous number.
