# Supplementary material for: The Dark and Gloomy Brain: Grey Matter Volume Alterations in Major Depressive Disorder–Fine-Grained Meta-Analyses
Source: Depress Anxiety. 2024 Mar 2;2024:6673522. doi: 10.1155/2024/6673522 (PMC11919126; doi:10.1155/2024/6673522)
Supplement: Supplementary Materials — Grey matter volume atrophy in the subgroup of patients with comorbid MDD and anxiety: an exploratory analysis. We conducted an exploratory analysis on the six experiments in which patients with comorbid MDD and one overt anxiety disorder showed GMV atrophy when compared with HC; we did not consider the cases of hypertrophy due to the even more exiguous number. [file 6673522.f1.zip › Romeo_Biondi_et.al_PRISMA2020_checklist.pdf]

PRISMA 2020 Checklist

Section and Topic	Item #	Checklist item	Location where item is reported
**TITLE**			
Title	1	The dark and gloomy brain: grey matter volume alterations in major depressive disorder – Two fine-grained meta-analyses	Romeo_Biondi_et.al_manuscript - page 1
**ABSTRACT**			
Abstract	2	**Background:** While the brain correlates of Major Depressive Disorder (MDD) have been extensively studied, there is no consensus conclusion so far. Various meta-analyses tried to determine the most consistent findings, but results are often discordant for grey matter volume (GMV) atrophy and hypertrophy. Applying rigorous and stringent inclusion criteria, and controlling for confounding factors, such as the presence of anxiety comorbidity, we carried out a novel meta-analysis on the existing literature to unveil MDD signatures.<br>**Methods:** A systematic literature search was performed up to January 2023. Seventy-three studies on MDD patients reporting GMV abnormalities were included in the first meta-analysis, for a total of 6167 patients and 6237 healthy controls (HC). To test the effects of anxiety comorbidity, we conducted a second meta-analysis, by adding to the original pure MDD sample a new cohort of MDD patients with comorbid anxiety disorders (308 patients *vs.* 342 HC).<br>An activation likelihood estimation (ALE) analysis and a coordinate-based mapping approach were used to identify common brain structural alterations among patients.<br>**Results:** The pure MDD sample exhibited an atrophic region centered in the left insula as well as hypertrophy in the bilateral amygdala and parahippocampal gyri. When we added patients with comorbid anxiety to the original sample, bilateral insula atrophy emerged, whereas the hypertrophy results were not replicated.<br>**Conclusions:** Our findings revealed important structural dysfunctions in pure MDD patients, in particular considering the insula key role in the sensory input integration to the emotional processing in the limbic system. Additionally, the amygdala and parahippocampus gyrus hypertrophy may depend on MDD functional over-activation to emotional stimuli, thus explaining patients' most relevant symptoms, including rumination and overactive self-referential thinking. Conversely, the presence of anxiety comorbidity produced effects not specifically attributable to MDD, thus revealing the importance of strict inclusion criteria for participant enrollment.	- page 2
**INTRODUCTION**			
Rationale	3	Some meta-analyses on the increasing body of literature on whole-brain VBM studies have been published in the last decade, with the aim to sum up the neural underpinnings of major depressive disorder (MDD). However, it should be pointed out that the abovementioned most results come from heterogeneous studies (e.g., including pharmacologically treated / drug free or first episode patients). Moreover, in some instances, broader selection criteria have been used: there are cases in which papers performing small volume correction or cases where other factors remained confused, such as the lack of distinction between grey matter density or concentration and GMV. Another issue worthy of attention is the presence of clinical samples with a spurious diagnostic profile, due to the inclusion in the MDD experimental group, for example, of remitted patients, subjects with a treatment-refractory condition, individuals suffering from secondary depression or who manifest subthreshold depressive symptoms. A particularly critical point is the frequent non-exclusion of studies investigating MDD patients with overt anxiety comorbidity. Anxiety and depression are distinct pathologies, characterized by specific diagnostic criteria and different clinical and pharmacological treatments. As a consequence, anxiety disorders might have a specific role in affecting brain structures when co-occurring with MDD, and they should be treated like any other comorbid pathologies.	- pages 3,4
Objectives	4	The main aim of this work is to contribute to shedding light on the intriguing and still not fully elucidated pathophysiology of MDD disorder, with a particular focus on the effects of co-occurrence of anxiety on brain anatomy.	- pages 4,5
**METHODS**			
Eligibility criteria	5	Studies were included if they met the following inclusion criteria: 1) they were in published in peer-reviewed journals in English; 2) they compared MDD patients with healthy controls (HC); 3) they reported GMV abnormalities using sMRI (studies with no	- pages 5,6

Section and Topic	Item #	Checklist item	Location where item is reported
		significant results were excluded, as well as studies investigating GM concentration or density); 4) they performed whole-brain VBM analysis (nor ROI or Small Volume Correction analyses were included); 5) they reported stereotactic coordinates in MNI or Talairach space (when the coordinates or the direction of the contrast between patients and HC were not clearly reported, we contacted the corresponding authors; in case of no response, these papers were excluded); 6) they did not investigate any kind of depression different from MDD (e.g., subthreshold depression, secondary depression, peri-partum/post-partum depression, psychotic or bipolar depression, dysthymic disorder or premenstrual dysphoric disorder; we also excluded patients in remission or in euthymic state, and those who were treatment-resistant); 7) they included patients with no overt physical, neurological or psychic comorbidities. To ensure results belonging to a "pure" MDD sample, we firstly excluded patients with any comorbid anxiety disorders -but not those showing anxious symptoms. After that, we retrieved these discarded works, to look at the effect produced by adding patients with both pathologies to the original sample.<br>To achieve maximum research coverage and a sample as representative as possible of the clinical population, we did not impose limitations on age (although 95% of works included in the final pool analyzed participants whose age was above 18 and below 65) or medication status. We also included studies performing different subgroup comparisons, as long as patient samples were not overlapping. Finally, in the case of longitudinal clinical trials, we considered only baseline pre-treatment (e.g., Electroconvulsive therapy) results.	
Information sources	6	We carried out a systematic search on both PubMed databases. In addition, we carefully inspected the reference lists of the most recent meta-analyses.	- page 5
Search strategy	7	The keywords used for the literature search were: 1) (major depression OR major depressive disorder OR MDD) AND (voxel) AND (morphometry), 2) (major depression OR major depressive disorder OR MDD) AND (structural MRI OR sMRI), 3) (major depression OR major depressive disorder OR MDD) AND (gray matter volume OR grey matter volume). The procedure returned 2111 records. Additionally, we inspected the complete reference lists of the previous meta-analyses on the matter.	- page 5
Selection process	8	Studies were included if they met the following inclusion criteria: 1) they were in published in peer-reviewed journals in English; 2) they compared MDD patients with healthy controls (HC); 3) they reported GMV abnormalities using sMRI (studies with no significant results were excluded, as well as studies investigating GM concentration or density); 4) they performed whole-brain VBM analysis (nor ROI or Small Volume Correction analyses were included); 5) they reported stereotactic coordinates in MNI or Talairach space (when the coordinates or the direction of the contrast between patients and HC were not clearly reported, we contacted the corresponding authors; in case of no response, these papers were excluded); 6) they did not investigate any kind of depression different from MDD (e.g., subthreshold depression, secondary depression, peri-partum/post-partum depression, psychotic or bipolar depression, dysthymic disorder or premenstrual dysphoric disorder; we also excluded patients in remission or in euthymic state, and those who were treatment-resistant); 7) they included patients with no overt physical, neurological or psychic comorbidities. To ensure results belonging to a "pure" MDD sample, we firstly excluded patients with any comorbid anxiety disorders -but not those showing anxious symptoms. After that, we retrieved these discarded works, to look at the effect produced by adding patients with both pathologies to the original sample.<br>To achieve maximum research coverage and a sample as representative as possible of the clinical population, we did not impose limitations on age (although 95% of works included in the final pool analyzed participants whose age was above 18 and below 65) or medication status. We also included studies performing different subgroup comparisons, as long as patient samples were not overlapping. Finally, in the case of longitudinal clinical trials, we considered only baseline pre-treatment (e.g., Electroconvulsive therapy) results. The inclusion process is summarized in the following flowchart (Figure 1).	- page 5,6
Data collection process	9	Three authors visually inspected "methods" and "results" paragraphs of each article, working independently. At the end of the procedure, the authors compared results and fixed the few doubts on critical papers. Data were manually extracted from the articles that fulfilled the inclusion criteria. Data extraction has been done independently, and the resulting file has been compared before running statistical analyses.	- page 6
Data items	10a	The final pool of works merged into the meta-analysis on the pure MDD sample consisted of 73 studies, listed in Table 1,	Table 1

Section and Topic	Item #	Checklist item	Location where item is reported
		from which we extracted data for a total of 6167 patients and 6237 HC. Notably, the number of healthy individuals here reported was obtained by counting them only once in case of studies performing different patients' subgroup comparisons with the same HC group [eg.,49].<br>Selected studies reported either decreases or increases in GMV, or both in some cases. In particular, 67 papers found GMV atrophy in patients with respect to HC, and six of them [49,75,78,82,85,98] found this result in both the comparisons they performed between two different subgroups of patients *vs*. the same HC group; thus, the total amount of contrasts for GMV atrophy was 73. In the case of GMV hypertrophy in patients compared to HC, 29 papers (23 of which also found atrophy, six hypertrophy only) returned this result; no different subgroup comparisons were performed, thus the final number of included contrasts was 29.<br>To test the effects of anxiety comorbidity, we retrieved the studies based on samples with dual diagnosis (n= 6) and an additional subsample affected by anxious comorbidity present in Qi et al. (2014) [50] previously discarded (Table 1). Therefore, we conducted the second meta-analysis adding to the original pure MDD sample a new cohort of 308 MDD patients with comorbid anxiety, and 342 HC, therefore including six further contrasts for GMV atrophy (n=79), and two for GMV hypertrophy (n=31). Therefore, this overall group, now labelled "MDD+A", consisted of 6475 patients and 6579 healthy individuals.	
	10b	When the coordinates or the direction of the contrast between patients and HC were not clearly reported, we contacted the corresponding authors; in case of no response, these papers were excluded	- page 5
Study risk of bias assessment	11	We carried out all the procedure manually, working as independent reviewers. All the results converged.	- page 6
Effect measures	12	As these are coordinate-based meta-analyses, we reported the minimum cluster size for considering a region statistically significant (in mm^3), the region of convergence size (in mm^3) and the center of the region (in MNI X,Y,Z coordinates, and the corresponding Brodmann Area(s)), the number of peak coordinates, the maximum ALE value, the p and z values of each peak, and the brain region (in MNI X,Y,Z coordinates, and the corresponding BA(s)).	- page 7
Synthesis methods	13a	As these are coordinate-based meta-analyses, we rigorously apply inclusion criteria to decide which studies were eligible, in particular considering our inclusion criteria. Then, the activation likelihood estimation (ALE) method [112] was performed under the software to quantitatively assess the inter-study concordance.	- page 5-7
	13b	Before running statistics, we converted all Talairach coordinates in the corresponding MNI coordinates, using the *convert foci option* interface in Ginger ALE software.	- page 7
	13c	We manually completed the Table that summarized the results of both meta-analysis 1 and 2.	Table 2, Table 3, Table 4, Table s1
	13d	In compliance with the guidelines by Müller and colleagues (2018) [109], statistical analyses were run via GingerALE software version 3.0.2 [110,111]. We carried out two different meta-analyses, the first one on the pure MDD sample and the second one on the MDD+A sample. Both of them were in turn subdivided into two meta-analyses performed separately, one for GMV atrophy and the other for GMV hypertrophy of patients compared to HC.<br>To weight studies contributions, GingerALE uses sample sizes and coordinates, which must be expressed in the same stereotactic space; therefore, the first step consisted in converting Talairach coordinates into MNI space using the convert foci option provided in the GingerALE interface. Then, the activation likelihood estimation (ALE) method [112] was performed under the software to quantitatively assess the inter-study concordance. ALE approach assesses spatial convergence of reported coordinates across the experiments against the null hypothesis that findings follow a random spatial distribution. The coordinates, or foci, are treated as three-dimensional Gaussian probability distributions centered at the given coordinates to generate per-experiment modeled atrophy/hypertrophy maps, which are subsequently joined in a union map [110,113]. For each Gaussian distribution, the algorithm derives full-width half-maximum by considering the sample size of every single study.	- page 7

PRISMA 2020 Checklist

Section and Topic	Item #	Checklist item	Location where item is reported
		Finally, ALE tests for above-chance spatial convergence through a range of available thresholding options. In our meta-analyses, we set a statistical ALE map threshold for significance using cluster-level family-wise error (FWE) correction at p<0.05 (5000 permutations), with cluster-forming threshold of p<0.01. Forasmuch as each coordinate referred to the contrast between two groups (patients vs. healthy controls), the analysis relied on the n of the smaller of the two samples to yield a more conservative activation likelihood estimation [114].	
	13e	To ensure results belonging to a “pure” MDD sample, we firstly excluded patients with any comorbid anxiety disorders -but not those showing anxious symptoms. After that, we retrieved these discarded works, to look at the effect produced by adding patients with both pathologies to the original sample.	- page 5
	13f	To ensure results belonging to a “pure” MDD sample, we firstly excluded patients with any comorbid anxiety disorders -but not those showing anxious symptoms. After that, we retrieved these discarded works, to look at the effect produced by adding patients with both pathologies to the original sample	- page 5
Reporting bias assessment	14	No missing results in our synthesis.	
Certainty assessment	15	In our meta-analyses, we set a statistical ALE map threshold for significance using cluster-level family-wise error (FWE) correction at p<0.05 (5000 permutations), with cluster-forming threshold of p<0.01. Forasmuch as each coordinate referred to the contrast between two groups (patients vs. healthy controls), the analysis relied on the n of the smaller of the two samples to yield a more conservative activation likelihood estimation.	- page 7
**RESULTS**			
Study selection	16a	Selected studies reported either decreases or increases in GMV, or both in some cases. In particular, 67 papers found GMV atrophy in patients with respect to HC, and six of them [49,75,78,82,85,98] found this result in both the comparisons they performed between two different subgroups of patients vs. the same HC group; thus, the total amount of contrasts for GMV atrophy was 73. In the case of GMV hypertrophy in patients compared to HC, 29 papers (23 of which also found atrophy, six hypertrophy only) returned this result; no different subgroup comparisons were performed, thus the final number of included contrasts was 29.<br>To test the effects of anxiety comorbidity, we retrieved the studies based on samples with dual diagnosis (n= 6) and an additional subsample affected by anxious comorbidity present in Qi et al. (2014) [50] previously discarded (Table 1). Therefore, we conducted the second meta-analysis adding to the original pure MDD sample a new cohort of 308 MDD patients with comorbid anxiety, and 342 HC, therefore including six further contrasts for GMV atrophy (n=79), and two for GMV hypertrophy (n=31). Therefore, this overall group, now labelled “MDD+A”, consisted of 6475 patients and 6579 healthy individuals.	Figure 1<br>- pages 6,7
	16b	From the starting literature pool (n=2111) we obtained by entering our keywords in PubMed, we excluded a total of 2038 studies, among which: 301 records were meta-analyses/reviews; 99 records were excluded basing on title/abstract screening (e.g., they were single-case studies, post-mortem studies, animal studies, etc…); 420 records had only healthy individuals or a clinical population different from MDD as the sample of interest; 181 records did not include a healthy control group; 89 records investigated a form of depression different from MDD (e.g., subthreshold depression, secondary depression, peri-partum/post-partum depression, psychotic or bipolar depression, dysthymic disorder or premenstrual dysphoric disorder, etc…); 73 records included MDD patients with an overt physical (e.g., diabetes), neurological (e.g., epilepsy) or psychic (e.g., eating disorder) comorbidities; 828 records did not perform a whole-brain VBM analysis comparing MDD patiens vs HC, but for example, they performed ROI or Small Volume Correction analyses; 12 records did not report the coordinates output or did not specify the coordinate system (e.g., MNI, Talairach); 35 records found not significant results or reported unsuitable contrasts (e.g., they .did not specify wether the areas they found altered were atrophic or hypertrophic).	Figure 1
Study characteristics	17	All Studies included in the our meta-analysis were listed in Table 1. All these studies satisfied inclusion criteria (see point #5).	Table 1
Risk of bias in	18	As we carried out two coordinate-based meta-analyses, there were no risk of bias in studies.	

Section and Topic	Item #	Checklist item	Location where item is reported
studies			
Results of individual studies	19	Here we copied the coordinate files divided between atrophy and ipertrophy, used for statistical analyses in Ginger ALE software.<br><br>**Pure MDD atrophy MNI coordinate file:**<br>Wagner et al.,2008:<br>-30.89 31.23 39.91<br>-.37 8.85 -28.99<br><br>Leung et al.,2009:<br>42.26 43.34 16.18<br>-52.25 -54.55 42.04<br>36.27 19.19 -35.12<br>63.07 14.88 32.09<br>1 -57.5 64.96<br>-53.89 -46.7 -.17<br>-11.08 -29.45 55.67<br><br>Mak et al.,2009:<br>19 43 13<br>66 11 44<br>13 -18 59<br>31 21 -31<br>-51 -46 -4<br>-50 -60 36<br>-1 -67 60<br><br>Zou et al.,2009:<br>-23.93 -19.23 -17.96<br>41.82 -12.49 -29.84<br><br>Cheng et al.,2010:<br>19 1 62<br><br>Hwang et al.,2010:<br>41.97 6.19 -13.76<br>17.7 -54.15 19.52<br>-17.95 -54.28 20.14<br><br>Scheuerecker et al.,2010:<br>-22 28 -21<br>-47 14 13<br>-63 -50 -4<br>64 -15 17<br>-35 -76 40<br>-65 -44 19<br>-49 -11 38	Authors' personal notebook

Section and Topic	Item #	Checklist item	Location where item is reported
		-51 -24 14<br>44 -80 -11<br>-51 24 -1<br>-58 -63 -14<br><br>Salvadore et al.,2011:<br>-49.6 28.57 12.48<br>-51.92 42.73 3.28<br>-38.67 42.72 25.46<br>56.19 46.92 7.74<br>44.5 48.33 23.49<br>32.41 50.03 7.84<br><br>Ma et al.,2012:<br>61 -34 -3<br><br>Wang et al.,2012:<br>45 -9 -39<br>-39 -6 -9<br>51 6 -21<br><br>Grieve et al.,2013:<br>-53 32 21<br>54 38 12<br>-47 15 18<br>48 52 -14<br>4 20 -29<br>-20 50 -12<br>15 47 -21<br>-14 56 5<br>17 52 13<br>-21 14 42<br>6 45 20<br>5 -27 45<br>-3 -38 24<br>-5 -69 38<br>6 -57 24<br>-9 11 54<br>56 -8 50<br>-60 -7 42<br>-45 -60 -21<br>50 -54 -17<br>50 -30 -21<br>-54 -34 -11<br>-51 -54 6<br>46 -22 0<br>-45 -25 40<br>54 -23 48	

Section and Topic	Item #	Checklist item	Location where item is reported
		-39 -48 36<br>33 -61 42<br>-5 -78 26<br>2 -92 -12<br>-32 -82 10<br>26 -70 25<br>32 -81 -5<br>-8 -4 13<br>-27 -13 -3<br>-9 9 8<br>-57 -57 -32<br>52 -54 -30<br>28 -63 -48<br>-28 -63 -45<br>-27 -69 -36<br><br>Chaney et al.,2014:<br>-9 56 12<br>-54 36 -8<br><br>Guo et al.,2014:<br>64.5 -54 -12<br>-43.5 -60 39<br><br>Jung et al.,2014:<br>-38 9 0<br>38 20 -3<br>21 45 33<br>-21 33 43<br>48 8 28<br>8 53 -21<br>18 -7 -12<br>33 -3 1<br>-27 -85 -21<br><br>Kong et al.,2014:<br>37 40 8<br>-36 19 42<br><br>Lai et al.,2014:<br>14.79 14.52 52.73<br>-13.7 19.16 67<br>-26.06 20.93 57.01<br>18.88 8.42 52.88<br>-42.94 7.21 6.23<br><br>Modinos et al.,2014:<br>0 27 -14	

Section and Topic	Item #	Checklist item	Location where item is reported
		9 30 -15<br>-8 24 -12<br>-48 -48 48<br>54 -14 -21<br>45 -51 54<br>-8 44 45<br><br>Nakano et al.,2014:<br>18 68 16<br>24 62 -2<br><br>Peng et al.,2014:<br>60 -53 -8<br><br>Peng et al.,2014:<br>-35 18 9<br><br>Qi et al.,2014:<br>-38 17 15<br><br>Cai et al.,2015:<br>26 19 -24<br><br>Dannlowski et al.,2015:<br>14 -6 -27<br>38 22 -5<br>-30 -72 -5<br>3 -10 43<br>-66 -27 12<br>4 -25 6<br><br>Fang et al.,2015:<br>-46.5 -10.5 -34.5<br>-43.5 22.5 7.5<br>-3 -72 16.5<br>6 -13 51<br>36 -31 -16.5<br>49.5 -7.5 -27<br>16.5 -97.5 16.5<br><br>Lai et al.,2015:<br>3.92 57.05 -16.97<br>-6.3 53.04 -16.73<br>18.18 62.88 -23.34<br>54.92 11.85 -26.2<br>-27.19 -83.91 -46.54<br>13.66 -64.06 -55.65	

Section and Topic	Item #	Checklist item	Location where item is reported
		Vasic et al.,2015:<br>65 1 -9<br>57 -48 21<br>-17 1 67<br>30 39 43<br>57 -42 -5<br>4 64 12<br><br>Watanabe et al.,2015:<br>13.92 10.76 -9.25<br>23.76 23.64 1.63<br>18.19 24.61 -10.69<br>-11.99 11.84 -7.79<br>-10.73 20.85 8.1<br>-9.77 25.44 -.21<br>-50.93 15.75 -9.76<br>-58.55 15.31 -14.07<br>-47.65 11.71 -7.17<br>-26.33 72 -17.99<br>-30.55 71.65 -10.03<br><br>Yang et al.,2015:<br>-25 -64 -4<br><br>Yang et al.,2015:<br>-2 43 29<br>-23 -65 -7<br>-5 53 28<br><br>Opel et al.,2016:<br>45 -13 16<br>-40 5 12<br>-69 -18 -8<br>26 24 3<br>-27 -57 -2<br>18 -73 45<br>34 -69 -6<br>-10 -31 33<br><br>Qiu et al.,2016:<br>15 5 43<br><br>Shen et al.,2016:<br>24 -61.5 27<br>30 -13.5 -19.5<br>19.5 13.5 -34.5<br>-1.5 -37.5 -12<br>-9 -66 -43.5	

Section and Topic	Item #	Checklist item	Location where item is reported
		-22.5 -33 -43.5<br><br>Wang et al.,2016:<br>-16 -6 -16<br>48 12 34<br><br>Igata et al.,2017:<br>-44 12 -7<br>-54 14 -3<br>-48 24 -25<br><br>Yang et al.,2017:<br>47 53 -8<br>35 51 4<br>-30 54 21<br>-17 64 20<br>46 10 30<br>30 -9 63<br>23 -75 -8<br>-46 0 33<br>47 -1 -6<br>47 -39 57<br>-36 -51 -24<br><br>Zhao et al.,2017:<br>-3 36 -30<br>-26 0 -3<br>27 3 -2<br>-5 -15 3<br>-39 -19 61<br>34 -22 63<br>-2 -73 -9<br><br>Zhuo et al.,2017:<br>-25.5 4.5 -21<br><br>Chang et al.,2018:<br>2 41 -17<br>41 -80 14<br>-29 -54 -11<br>-27 54 3<br>-38 2 11<br>51 -57 23<br>5 -84 25<br>-30 27 39<br>15 3 72<br><br>Chen et al.,2018:	

Section and Topic	Item #	Checklist item	Location where item is reported
		27 -11 -12<br>-3 41 24<br>-12 24 47<br><br>Lu et al.,2018:<br>-24 -37.5 -10.5<br>31.5 -43.5 -13.5<br>60 -63 1.5<br><br>Zaremba et al.,2018:<br>34 36 28<br><br>Zhou et al.,2018:<br>19.5 -85.5 21<br><br>Hellewell et al.,2019:<br>-3 -6 9<br>42 -21 -7<br>-46 -27 -1<br>-37 35 -1<br>51 45 9<br>46 -11 51<br>-36 -87 6<br>-39 -57 -13<br>-49 -59 30<br>-44 -19 45<br>-2 -87 37<br>33 -70 47<br><br>Hellewell et al.,2019:<br>12 28 -28<br>48 -53 -18<br>-64 -39 -22<br>-52 32 24<br>25 64 8<br>43 -12 49<br>-32 -88 8<br>-44 -54 -17<br>-50 -56 24<br>-45 -11 52<br>-1 -75 46<br>34 -67 -55<br><br>Kandilarova et al.,2019:<br>-14 55 0<br><br>Li et al.,2019:<br>-3 -30 -12	

PRISMA 2020 Checklist

Section and Topic	Item #	Checklist item	Location where item is reported
		6 -33 -12<br>-18 -6 3<br>18 6 3<br>-12 -6 0<br>18 -9 0<br><br>Liu et al.,2019:<br>-18 -36 -12<br>35 5 51<br><br>Liu et al.,2019:<br>-17 -32 -12<br>18 -24 -15<br><br>Peng et al.,2019:<br>53 14 24<br>27 44 -9<br><br>Chen et al.,2020:<br>28 -64 -8<br>-46 -36 62<br><br>Liu et al.,2020:<br>-17 -21 -18<br><br>Liu et al.,2020:<br>-27 44 -16<br><br>Meng et al.,2020:<br>48 -6 -4.5<br><br>Nan et al.,2020:<br>22.5 15 -36<br><br>Yang et al.,2020:<br>41 30 0<br>0 33 -17<br>-38 17 -21<br>-50 -12 51<br>-21 -2 -17<br>18 -2 -17<br>-42 -17 11<br>-12 21 12<br><br>Yang et al.,2020:<br>0 33 -17<br>-38 17 -21<br>-50 -12 51	

Section and Topic	Item #	Checklist item	Location where item is reported
		-21 -2 -17<br>18 -2 -17<br>-42 -17 11<br><br>Zhang et al.,2020:<br>0 58.5 -10.5<br>-67.5 -22.5 -3.0<br><br>Jiang et al.,2021:<br>6 -69 30<br>9 -39 57<br><br>Liu et al.,2021:<br>6 12 3<br>-36 8 39<br><br>Ma et al.,2021:<br>14 53 20<br>5 56 26<br>17 57 5<br><br>Takamiya et al.,2021:<br>-64 -50 6<br>-60 -57 -4<br>-50 -62 -8<br>-38 -46 -14<br>-51 -58 -22<br>-50 -51 -8<br>-46 -50 -27<br>-22 -44 -14<br>-34 -62 -9<br>-51 -50 22<br>-56 -46 32<br>-58 -57 28<br>14 -86 -10<br>32 -100 -2<br>24 -93 -6<br>30 -86 -10<br>44 -75 -14<br>30 -75 -16<br>46 -58 -20<br><br>Zhang et al.,2021:<br>33 3 -52.5<br>-43.5 -82.5 -15<br>-6 -31.5 58.5<br>25.5 12 -37.5<br>45 -21 -30	

PRISMA 2020 Checklist

Section and Topic	Item #	Checklist item	Location where item is reported
		49.5 -66 -46.5<br><br>Zhou et al.,2021:<br>25.5 -66 -60<br>22.5 -9 10.5<br>61.5 -37.5 -16.5<br>-24 39 -12<br>-54 -52.5 -1.5<br>-24 -24 -7.5<br>9 13.5 -1.5<br>0 -55.5 40.5<br>-3 9 54<br>0 -40.5 52.5<br>54 -28.5 52.5<br>31.5 16.5 55.5<br><br>Kang et al.,2022:<br>31.5 -16.5 4.5<br><br>Li et al.,2022:<br>48 4.5 -27<br>56 -6 -11<br>-51 -3 -19.5<br>-6 -52.5 9<br>9 -43.5 10.5<br>-12 48 28.5<br><br>Liu et al.,2022:<br>46.5 9 51<br>52.5 -10.5 49.5<br><br>Lu et al.,2022:<br>31.5 26.5 4.5<br>-32.5 16.5 5.5<br>5.5 48.5 -23.5<br>-5.5 22.5 -23.5<br>36.5 51.5 2.5<br>-24.5 54.5 12.5<br>48.5 0.5 4.5<br>-51.5 0.5 4.5<br>23.5 56.5 13.5<br>-20.5 55.5 10.5<br>7.5 49.5 -23.5<br>-8.5 39.5 -26.5<br>8.5 47.5 0.5<br>-2.5 51.5 6.5<br>8.5 33.5 -10.5<br>-5.5 60.5 -13.5	

PRISMA 2020 Checklist

Section and Topic	Item #	Checklist item	Location where item is reported
		27.5 54.5 -10.5<br>-22.5 40.5 -10.5<br>54.5 36.5 1.5<br>-33.5 26.5 -0.5<br>29.5 16.5 -21.5<br>-49.5 10.5 3.5<br>56.5 -27.5 -24.5<br>3.5 45.5 4.5<br>-6.5 44.5 5.5<br>-2.5 26.5 32.5<br>-54.5 6.5 -0.5<br>48.5 -4.5 -13.5<br>52.5 -7.5 -22.5<br>-62.5 -32.5 -0.5<br>-58.5 -2.5 -14.5<br>-61.5 -31.5 -20.5<br>41.5 -9.5 45.5<br>-29.5 -11.5 53.5<br>54.5 -7.5 33.5<br>9.5 6.5 48.5<br>-9.5 11.5 47.5<br>1.5 8.5 -6.5<br>-1.5 9.5 -6.5<br>-35.5 -84.5 1.5<br>-45.5 -68.5 -13.5<br>36.5 -17.5 -29.5<br>-39.5 -39.5 -22.5<br>44.5 -58.5 44.5<br>30.5 17.5 1.5<br>-29.5 -11.5 2.5<br>3.5 9.5 -6.5<br>-1.5 8.5 -6.5<br>59.5 -57.5 28.5<br>8.5 -73.5 50.5<br>35.5 -15.5 -30.5<br>-61.5 -30.5 37.5<br>6.5 -60.5 -43.5<br>9.5 -64.5 -43.5<br>-6.5 -68.5 -29.5<br>-35.5 -67.5 -33.5<br>-4.5 -70.5 -30.5<br><br>Lu et al.,2022:<br>-12 3 -9<br><br>Sun et al.,2022:<br>7.5 15 7.5<br>-48 12 -9	

Section and Topic	Item #	Checklist item	Location where item is reported
		48 6 -7.5<br>6 15 22.5<br><br>Sun et al.,2022:<br>19.5 7.5 6<br>-19.5 0 7.5<br><br>Wang et al.,2022:<br>-46.5 10.5 -9<br>49.5 13.5 -4.5<br>0 9 24<br>13.5 -21 36<br>42 13.5 21<br>21 -61.5 42<br><br>Yang et al.,2022:<br>-34.5 31.5 34.5<br>-48 -1.5 -18<br>3 -73.5 -6<br>-54 3 1.5<br><br>Yu et al.,2022:<br>30 -30 56<br>6 -35 72<br>20 -24 66<br>-6 -24 42<br>8 -17 51<br>-14 50 -8<br>-14 41 -5<br>-6 44 -11<br><br>**MDD+A atrophy MNI coordinate file:**<br>Lai et al.,2010:<br>-1.86 17.71 22.59<br>-3.89 17.78 24.63<br>3.83 16.27 -21.75<br>44.79 40.56 -18.92<br>14.16 -42.17 -5.96<br>-14.4 -27.65 -0.05<br>-14.48 4.5 -11.12<br>28.36 -0.08 -21.51<br>46.79 14.04 -22.15<br>-4.21 -74.37 1.28<br>-10.5 -76.87 -14.7<br>14.09 -50.48 -11.74<br><br>Stratmann et al.,2014:<br>36 23 -5	

Section and Topic	Item #	Checklist item	Location where item is reported
		-24 -72 45<br>-60 -1 -3<br>56 -7 -12<br>-27 -31 -20<br><br>Harada et al.,2016:<br>0 37.5 -13.5<br>-28.5 34.5 -16.6<br>45 42 6<br>46.5 18 -3<br>-43.5 -61.5 19.5<br>60 -22.5 -4.5<br><br>Yang et al.,2017:<br>-4.5 9 64.5<br>-43.5 -7.5 -3<br>39 0 1.5<br>-27 6 -9<br>33 0 1.5<br>24 6 -15<br>22.5 4.5 -19.5<br>-12 -51 -9<br>-52.5 6 -58.5<br><br>Lu et al.,2019:<br>-17 -66 57<br><br>Zhang et al.,2021:<br>42 7.5 -16.5<br>19.5 -78 -18<br><br>**Pure MDD hypertrophy MNI coordinate file:**<br>Leung et al.,2009:<br>-1.85 -42.94 14.26<br><br>Hwang et al.,2010:<br>16.48 -102.28 -1.47<br>36.51 -77.46 -35.64<br><br>Scheuerecker et al.,2010:<br>13 -50 -24<br>42 -82 -31<br>53 -5 17<br>21 -9 56<br>18 -61 47<br>-35 39 11<br>23 1 -17	

Section and Topic	Item #	Checklist item	Location where item is reported
		Amico et al.,2011:<br>66 -25 -7<br><br>Chaney et al.,2014:<br>24 -88 13<br>46 -33 -12<br>50 -76 -9<br>-12 -6 -12<br><br>Kong et al.,2014:<br>-5 -14 11<br>43 0 3<br><br>Peng et al.,2014:<br>39 -39 60<br><br>Qiu et al.,2014:<br>-3 -27 61<br>-3 42 51<br>-12 -91 28<br>-18 -24 5<br>12 -90 30<br>16 -19 6<br><br>Yang et al.,2015:<br>-3 -48 8<br><br>Yang et al.,2015:<br>-3 -45 5<br>-64 8 20<br><br>Chen et al.,2016:<br>45 -33 56<br><br>Shen et al.,2016:<br>49.5 -64.5 24<br>42 -84 28.5<br><br>Zhao et al.,2017:<br>39 14 -30<br><br>Chen et al.,2018:<br>-18 -20 0<br><br>Lu et al.,2018:<br>-15 27 10.5<br>43.5 -66 -19.5<br>-52.5 -51 -25.5	

Section and Topic	Item #	Checklist item	Location where item is reported
		-10.5 -88.5 -19.5<br><br>Gong et al.,2019:<br>32 6 -2<br>-30 5 -7<br><br>Li et al.,2019:<br>-21 0 -15<br>21 3 -15<br>-15 -9 -18<br>27 -6 -18<br>-12 0 -18<br>18 -6 -18<br>24 -39 -18<br><br>Liu et al.,2019:<br>Subjects=21<br>20 -69 33<br><br>Peng et al.,2019:<br>-25 -90 19<br>8 -53 -22<br><br>Peng et al.,2019:<br>8 -53 -22<br><br>Straub et al.,2019:<br>45 18 51<br>-14 -18 20<br><br>Liu et al.,2020:<br>21 -69 32<br><br>Nan et al.,2020:<br>37.5 -75 30<br><br>Yang et al.,2020:<br>0 -101 -5<br><br>Liu et al.,2021:<br>33 -38 -17<br>45 -3 -15<br>-14 -61 1<br><br>Liu et al.,2021:<br>Subjects=335<br>-46 6 -20<br>46 3 -20	

Section and Topic	Item #	Checklist item	Location where item is reported
		Zhang et al.,2021:<br>-19.5 7.5 43.5<br>-61.5 -28.5 45<br><br>Sun et al.,2022:<br>15 -102 -12<br><br>Zhang et al.,2022:<br>18 -33 2<br><br>**MDD+A hypertrophy MNI coordinate file:**<br>Qi et al.,2014:<br>-44 -14 59<br>-6 -33 12<br>0 -8 -18<br>-20 -77 29<br>-2 66 -6<br>-48 -20 18<br>27 -24 71<br>59 -6 50<br>14 -45 77<br><br>Lu et al.,2019:<br>30 -78 27	
Results of syntheses	20a	No risk of bias among contributing studies.	
	20b	The first meta-analysis was conducted on 73 contrasts (derived from 67 papers) that included 407 foci and compared 5509 pure MDD patients showing GMV atrophy versus 6618 HC (here the healthy individuals were counted twice in case of contrasts between two different subgroups of patients and the same HC group, as each contrast is taken into account separately). The minimum size for a cluster to be considered statistically significant was 2000 mm3. Our results revealed a region of convergence of 3064 mm3 centered in the left Insula (MNI coordinates: X= -47.3, Y= 9.1, Z= -1.7), with four peaks, three of which localized in the left Insula (corresponding to Brodmann area [BA] 13) and one in the left Superior Temporal Gyrus (BA 22). The maximum ALE value (0.0341, p< 0.00000001; z= 5.54) was found within the left Insula (MNI coordinates: X= -46, Y= 12, Z= -8). Table 2 provides a summary of all significant results. Figure 2 shows that the significant cluster was lateralized in the left hemisphere and that it included the Insula and the Superior Temporal Gyrus.<br><br>Secondly, we meta-analyzed 29 contrasts (derived from 29 papers), for a total of 63 foci, in which 2188 pure MDD patients had higher GMV than 3072 HC. The chosen minimum cluster size to be statistically significant was 1680 mm3, and two regions converged above this threshold. The first cluster of 1696 mm3 was centered in the left Parahippocampal Gyrus (MNI coordinates: X= -14.7, Y= -4.2, Z= -15.5) with three peaks, all of them belonging to the left Parahippocampal Gyrus (BA 28/BA 34). The maximum ALE value (0.0121, p< 0.00006059; z= 3.84) was found within the left Parahippocampal Gyrus (MNI coordinates: X= -14, Y= -8, Z= -16). The second cluster of 1688 mm3 was centered in the right Parahippocampal Gyrus (MNI coordinates: X= 22.4, Y= -1.3, Z= -16.8) with three peaks (right BA 28/BA 34 and right Amygdala), and the maximum ALE value (0.0156, p< 0.00000596; z = 4.38) was found within the right Parahippocampal Gyrus (MNI coordinates: X= 22, Y= 2, Z= -16). A summary of all significant results is provided in Table 3, whereas Figure 3 shows the significant clusters including the left and right Parahippocampal Gyri and Amygdala.	- pages 8-10

Section and Topic	Item #	Checklist item	Location where item is reported
		A meta-analysis was also conducted on 79 contrasts (derived from 73 papers) including 442 foci and comparing 5797 MDD+A patients showing reduced GMV versus 6960 HC (here the healthy individuals were counted twice in case of contrasts between two different subgroups of patients and the same HC group, as each contrast is taken into account separately). The minimum size for a cluster to be considered statistically significant was 2304 mm3. The results showed two regions of convergence. The first cluster, 4216 mm3 in size, was centered in the right Insula (MNI coordinates: X= 42.6, Y= 10.8, Z= -7.1) and had eight peaks, distributed among the Insula (BA 13), Superior Temporal Gyrus (BA 21), Temporal lobe (BA 38) and Claustrum, all in the right hemisphere. The maximum ALE value (0.027, p< 0.00000166; z= 4.65) was localized in the right Insula (MNI coordinates: X= 38, Y= 22, Z= -4). The second cluster was 2952 mm3 and centered in the left Insula (MNI coordinates: X= -47.9, Y= 8.8, Z= -1.8) with four peaks, three of which localized in the left Insula (BA 13) and one in the left Superior Temporal Gyrus (BA 22). The maximum ALE value (0.0341, p< 0.00000002; z= 5.47) was found in the left Insula (MNI coordinates: X= -46, Y= 12, Z= -8). Table 4 lists all significant peaks and Figure 4 displays the significant clusters.<br>A second meta-analysis on 2238 MDD+A patients showing higher GMV compared to 3148 HC was conducted, comprising 31 contrasts (derived from 31 papers) for a total of 73 foci, but in this case no statistically significant clusters were found.	
	20c	No evidence of heterogeneity among study results.	
	20d	When we added patients with comorbid anxiety to our pure MDD sample, bilateral insula atrophy emerged. Interestingly, as gingerALE software reveals which studies contribute to the results, we noticed that one-third of the studies contributing to the right hub included patients with comorbidities. In other words, although the patients with comorbidities showing atrophy were 1/19 of the pure ones (288/5509), their contribution was proportionally very large to the right insula result, a hub that did not spontaneously emerge in the pure MDD sample. Differently, only one study contributing to the left hub belonged to the MDD+A sample. Considering these ratios, and the fact that the left insula was the main atrophic result of our pure MDD meta-analysis, we suggest that the left insula might be a more reliable and peculiar hub of major depressive patients, whereas the right homologous might be less specific, or characteristic of a clinical population which, together with depression, also suffers from an anxiety disorder.	
Reporting biases	21	No missing results in our datasets.	
Certainty of evidence	22	No assessments of certainty (or confidence) in the body of evidence for our analyses.	
**DISCUSSION**			
Discussion	23a	The present work represents, to the best of our knowledge, the most complete and updated coordinate-based meta-analysis (CBMA) on MDD.<br>The analysis of our pure MDD sample and similar healthy adults revealed two important findings: depressive patients exhibited both GMV atrophy and hypertrophy. Considering the GMV loss, our results on more than 5500 pure MDD patients revealed an atrophic region centered in the left insula, a key region involved in emotional responses [128], language, sensory-motor, decision-making, salience, attentional processing [e.g., 129,130,131] and interoception; in particular, the anterior portion of insula is critical for processing the emotional component of interoception awareness, mainly depending on its connection with limbic regions [135,136]. Numerous previous whole-brain meta-analyses [7-24] found GMV atrophy occurring in depressed patients, but some of their results were discordant; this might depend on the application of less stringent inclusion criteria, such as the inclusion of spurious samples with anxiety comorbidity. Indeed, when we added patients with comorbid anxiety to our pure MDD sample, bilateral insula atrophy emerged. Although the patients with comorbidities showing atrophy were 1/19 of the pure ones (288/5509), their contribution was proportionally very large to the right insula result, a hub that did not spontaneously emerge in the pure MDD sample. We suggest that the left insula might be a more reliable and peculiar hub of major depressive patients, whereas the right homologous might be less specific, or characteristic of a clinical population which, together with depression, also suffers from an anxiety disorder.<br>We also found hypertrophy in bilateral amygdala and parahippocampal gyri in the pure MDD sample, two key areas involved in the circuitry via which emotions and memory functions have a modulatory effect on each other [144]. This result, although	- pages 10-15

Section and Topic	Item #	Checklist item	Location where item is reported
		still limited, is the newest and most promising one, given the recent and growing interest in structural hypertrophy. We wondered whether our hypertrophy results depend on drug response, but what emerged from a kind of *"post-hoc quality check"* of the GingerALE output is that most of the contributing studies included only drug-free patients. Contrary to what we expected, our result revealed that hypertrophy was not a secondary phenomenon to pharmacological treatment. Then, an highly speculative interpretation of our results is that patients' hypertrophy represents a biomarker of pure MDD, and not to their treatment response. In other words, some subcortical circuits, mainly involved in negative-valences emotional processing, are hyperstimulated by the disease symptoms, thus leading to an increased GMV. When considering the MDD+A meta-analysis, in which only 50 patients with anxiety comorbidity were added, the result obtained for the pure MDD sample was not replicated. As comorbidity patients usually undergo multiple drug treatments, as they are characterized by more severe clinical pictures, a possible interpretation is that the addition of spurious participants may obscure the effects that appear spontaneously in a group of pure ones. Past relevant studies from the ENIGMA MDD Consortium provided evidence of subcortical brain atrophy in a region close to our hub, i.e., the hippocampus [141,142,143]. Notably, these analyses were all carried out not following a whole-brain approach. In their recent review, Schmaal and colleagues found reduced hippocampal volume in MDD patients compared with controls, with a modest effect size [141] and hippocampus atrophy appeared related to long-lasting, persistent and recurring forms of MDD. Therefore, hippocampal atrophy appears related to later phases of MDD, rather than to premorbid vunerability factor [141]. In line with this evidence, the late structural atrophy may depend on abnormal functional activation of subcortical limbic regions during the early phases of the disorder.	
	23b	The present study has some limitations. Firstly, only six studies met all our inclusion criteria for MDD patients with anxiety comorbidities. Nevertheless, they led to important changes in our results. By adding future studies, it might be possible to disambiguate pure MDD brain correlates from those influenced also by anxiety comorbidity. Secondly, in our work we explored the drug status contribution only qualitatively, mainly because, in many cases, it was not possible to distinguish between drug-free patients and drug-naïve patients. Future studies may clarify this aspect, investigating results depending on pharmacological effects from those specific to MDD. Finally, GingerALE software grounds on a coordinate-based approach, and the dataset for atrophy and hypertrophy are considered in separate analysis. The incusion of studies with no significant results (n=24 out of 35 no results/unsuitable contrasts in Figure 1) is therefore not allowed.	- page 15
	23c	We identified no specific limitations in the review processes used.	
	23d	Increasing the sample size might not necessarily make the analysis statistically more powerful, as it might add a level of variance capable of modifying the results and their interpretation. In our specific case, we suggest that including data that makes the pure sample spurious may produce less reliable results. In particular, the presence of overt comorbidity between MDD and anxiety (unlike the occurrence of anxious symptoms only, a fact well-known from clinical practice) in the MDD+A meta-analyses may have produced effects not specifically attributable to major depression.<br>Our work highlights the importance of using rigorous and stringent inclusion criteria for participant enrollment, in particular considering MDD patients with anxious comorbidity.	- page 15
**OTHER INFORMATION**			
Registration and protocol	24a	These coordinate-based meta-analyses were not registered.	
	24b	A protocol was not prepared.	
	24c	No amendments to information provided at registration or in the protocol.	
Support	25	No financial support for the study.	
Competing interests	26	No competing interests of meta-analysis authors.	
Availability of data, code and other materials	27	The template data collection forms; data extracted from included studies; data used for all analyses; analytic code; any other materials used in the meta-analyses can be requested to the corresponding author upon justified request for academic purposes only.	

From: Page MJ, McKenzie JE, Bossuyt PM, Boutron I, Hoffmann TC, Mulrow CD, et al. The PRISMA 2020 statement: an updated guideline for reporting systematic reviews. BMJ 2021;372:n71. doi: 10.1136/bmj.n71
For more information, visit: http://www.prisma-statement.org/
