# Supplementary material for: The Dark and Gloomy Brain: Grey Matter Volume Alterations in Major Depressive Disorder–Fine-Grained Meta-Analyses
Source: Depress Anxiety. 2024 Mar 2;2024:6673522. doi: 10.1155/2024/6673522 (PMC11919126; doi:10.1155/2024/6673522)
Supplement: Supplementary Materials — Grey matter volume atrophy in the subgroup of patients with comorbid MDD and anxiety: an exploratory analysis. We conducted an exploratory analysis on the six experiments in which patients with comorbid MDD and one overt anxiety disorder showed GMV atrophy when compared with HC; we did not consider the cases of hypertrophy due to the even more exiguous number. [file 6673522.f1.zip › Romeo_Biondi_Supplementary_Materials.pdf]

Supplementary Materials for

The dark and gloomy brain: grey matter volume alterations in major depressive disorder – fine-grained meta-analyses

Zaira Romeo[1#], Margherita Biondi[1#], Leif Oltedal[2,3] and Chiara Spironelli[1,4*]

[1] Department of General Psychology, University of Padova, 35131 Padova, Italy

[2] Department of Clinical Medicine, University of Bergen, 5020 Bergen, Norway

[3] Mohn Medical Imaging and Visualization Centre, Department of Radiology, Haukeland University Hospital, 5021 Bergen, Norway

[4] Padova Neuroscience Center, University of Padova, 35131 Padova, Italy

* Correspondence: chiara.spironelli@unipd.it; Tel.: +39-049-827-6619

Grey matter volume atrophy in the subgroup of patients with comorbid MDD and anxiety: an exploratory analysis

We conducted an exploratory analysis on the six experiments in which patients with comorbid MDD and one overt anxiety disorder showed GMV atrophy when compared with HC; we did not consider the cases of hypertrophy due to the even more exiguous number. The number of included foci were 35. With a cluster size minimum threshold of 1648 mm^3, we found one region of convergence centered within the right Parahippocampal Gyrus (MNI coordinates: X= 25.1, Y= 3.2, Z= -19). This cluster presented only one peak corresponding to the right Subcallosal Gyrus (MNI coordinates: X= 24, Y= 4, Z= -20, BA 34), which had also the maximum ALE value (0.0103, <0.00003191; z= 4) (Table S1 and Figure S1).

Cluster	Anatomical label	BA	MNI coordinates			Size (mm^3)	ALE value	*p* value	Z score
			x	y	z				
1	Right Subcallosal Gyrus	34	24	4	-20	1648	0.010328273	<0.000032	3.998222

Table S1. Significant peak belonging to the cluster of GMV atrophy in the subgroup of patients with comorbid MDD and anxiety *vs.* healthy controls.

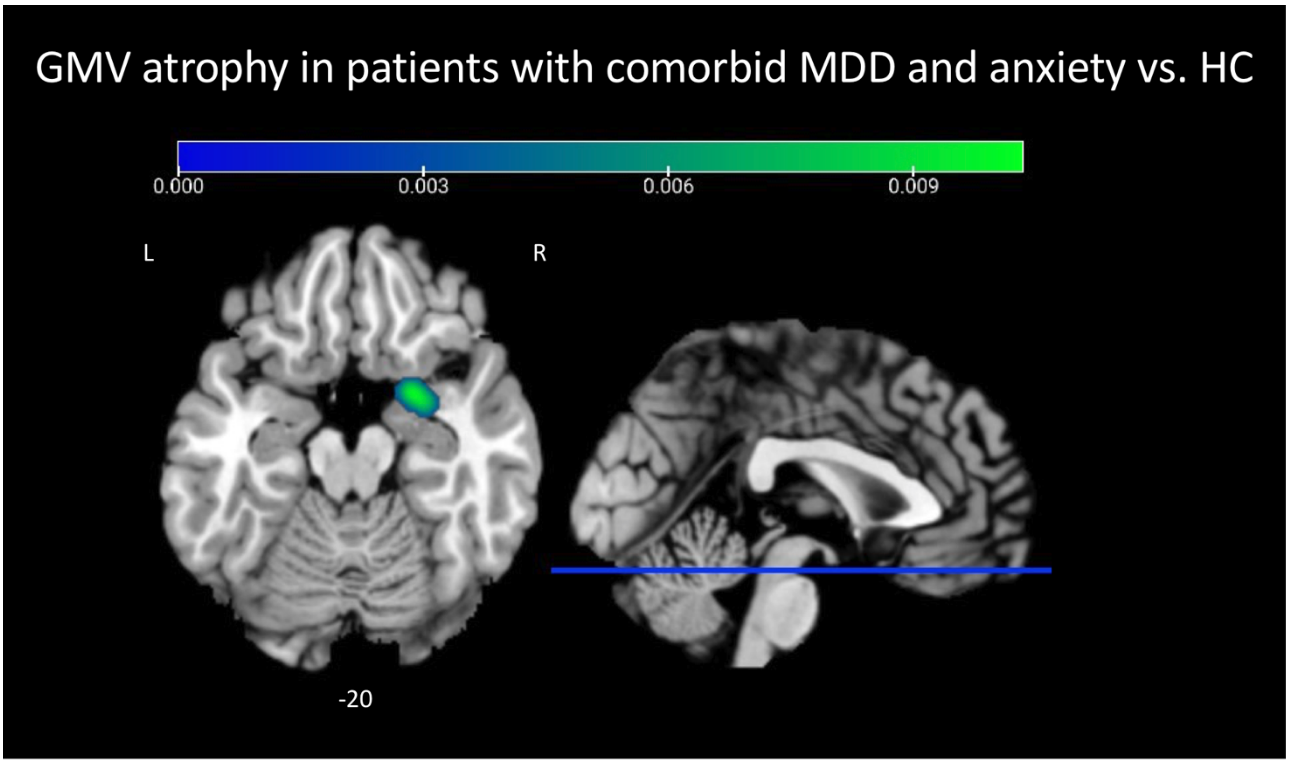

Figure S1. Results obtained from the meta-analysis focused on grey matter volume loss in the subgroup of patients with comorbid MDD and anxiety compared to HC.
